# Statistical analysis plan for the ‘Efficacy of Nitric Oxide in Stroke’ (ENOS) trial

**DOI:** 10.1111/ijs.12235

**Published:** 2014-03-03

**Authors:** Philip M W Bath, Aimee Houlton, Lisa Woodhouse, Nikola Sprigg, Joanna Wardlaw, Stuart Pocock, on behalf of the ENOS Trialists

**Affiliations:** 1 Stroke Trials Unit, Division of Clinical Neuroscience, University of NottinghamNottingham, UK; 2 Neuroimaging Sciences, University of EdinburghEdinburgh, UK; 3 Department of Medical Statistics, London School of Hygiene and Tropical MedicineLondon, UK

**Keywords:** acute stroke trial, blood pressure, glyceryl trinitrate, intracerebral hemorrhage, ischemic stroke, statistical analysis plan

## Abstract

High blood pressure is common during the acute phase of stroke and is associated with a poor outcome. However, the management of high blood pressure remains unclear. The ‘Efficacy of Nitric Oxide in Stroke’ trial tested whether transdermal glyceryl trinitrate, a nitric oxide donor that lowers blood pressure, is safe and effective in improving outcome after acute stroke. Efficacy of Nitric Oxide in Stroke is an international multicenter, prospective, randomized, single-blind, blinded endpoint trial, with funding from the UK Medical Research Council. Patients with acute ischemic stroke or intracerebral hemorrhage and systolic blood pressure 140–220 mmHg were randomized to glyceryl trinitrate or no glyceryl trinitrate and, where relevant, to continue or stop prestroke antihypertensive therapy. The primary outcome is shift in modified Rankin Scale at three-months. Patients or relatives gave written informed (proxy) consent, and all sites had research ethics approval. Analyses will be done by intention to treat. This paper and attachment describe the trial's statistical analysis plan, developed prior to unblinding of date. The statistical analysis plan contains design and methods for analyses, and unpopulated tables and figures for the two primary publications and some secondary publications. The database will be locked in late February 2014 in preparation for presentation of the results in May 2014. The data from the trial will improve the precision of the estimates of the overall treatment effects (efficacy and safety) of results from completed trials of blood pressure management in acute stroke, and provide the first large-scale randomized evidence on transdermal glyceryl trinitrate, and of continuing (vs. stopping) prestroke antihypertensive medications, in acute stroke.

High blood pressure (BP) is present in 70% or more of patients with acute ischemic stroke and intracerebral hemorrhage (ICH) 1. Affected patients have a worse outcome, whether judged as early recurrence, death within a few weeks, or combined death and dependency after several months 1–4. Lowering BP might therefore reduce these events and improve functional outcome providing that cerebral perfusion is not reduced in the presence of dysfunctional cerebral autoregulation. However, recent large trials have been inconsistent and inconclusive in their results 5,6.

Nitric oxide (NO) donors are candidate treatments for acute stroke: NO is a cerebral and systemic vasodilator, modulates vascular and neuronal function, and inhibits apoptosis 7.

Preclinical studies of cerebral ischemia found that NO donors reduce stroke lesion size and improve regional cerebral blood flow (CBF) and functional outcome 8. Five small clinical studies of NO donors have been performed, these involving a total of 208 patients with recent stroke.

Intravenous sodium nitroprusside reduced BP without altering CBF and exhibited antiplatelet effects (thereby precluding its use in ICH) 9. Four pilot trials of transdermal glyceryl trinitrate (GTN) found that it lowered BP by approximately 8%; did not alter platelet function (and so could be given in ICH); did not alter middle cerebral artery blood flow velocity or regional CBF; improved aortic vascular compliance; and could be given to patients with dysphagia 10–13. No safety concerns were present in these studies, and in one small trial, ultra-acute treatment with GTN was associated with an improved functional outcome 13,14.

On the basis of these preclinical and clinical data showing feasibility, tolerability and apparent safety of GTN, and the potential for efficacy, the large ‘Efficacy of Nitric Oxide in Stroke’ (ENOS) trial was started and is ongoing. ENOS is assessing, in a partial, factorial, prospective, randomized, single-blind, blinded-outcome design, whether to lower BP with GTN (vs. no GTN) and whether to continue (vs. stop) prestroke antihypertensive therapy. The trial commenced in 2001, and protocols for the main trial and an outline on the management of neuroimaging were published in 2006 and 2007, respectively 15,16. Several nontreatment-related and blinded analyses of the ENOS database have been published since the start of the trial 17–22. The independent Data Monitoring Committee have assessed the trial every six-months and on each occasion recommended that the trial should continue.

Prior to presentation of the primary analyses in 2014, two further publications are planned, the statistical analysis plan (SAP) and a detailed listing of baseline characteristics. The accompanying Supporting Information Appendix S1 details the SAP and is presented prior to locking of the trial database (expected in late February) so that analyses are not data driven or selectively reported 23. Unusually, this SAP includes not just information on the two primary publications (GTN vs. no GTN, and continue vs. stop prestroke antihypertensive medication) but also provides detailed information on the intended baseline characteristics publication and the first set of secondary publications. The SAP also informs much of the content of the final trial report to be submitted to the Medical Research Council/Efficacy and Mechanism Evaluation Programme (EME); the final report will be submitted in the third quarter of 2014 for publication in the *EME Journal*, part of the National Institute for Health research collection of peer-reviewed open access journals.

Importantly, the ENOS Trial Steering Committee have changed the original plan for the analysis of the primary outcome, as reported in the protocol (published in IJS) 15, from using an unadjusted binary ‘cut’ of the modified Rankin Scale (mRS 24, unadjusted comparison of mRS >2 between the treatment groups) to an adjusted ordinal analysis utilizing all seven levels of the mRS with adjustment for minimization variables. The change meant that the sample size could be reduced from 5000 patients to a minimum of 3500 patients assuming power of 90% and significance of 5%. The decision to change from dichotomous to polytomous analysis was not based on any interim analysis of the ENOS dataset; rather, it reflects the recognition that ordinal analyses are more efficient statistically (i.e. they provide improved statistical power for a given sample size) 25,26 as also shown for head injury trials 27. (The importance of this change is highlighted by recent trials that were technically neutral on their primary outcome when using a binary analysis but positive when analyzed secondarily using an ordinal analysis. 6,28) Similarly, adjusted analyses provide additional statistical power 29, are important if minimization is used during the process of randomization 30, and help address any minor imbalances present at baseline because of chance. As a result, these statistical approaches are likely to be more sensitive to any treatment effect and, as such, are recommended by the European Stroke Organization 31. The collection of all baseline data needed for covariate adjustment of the primary outcome should mean there is no need for imputation for missing data.

In the future, data from ENOS will be integrated into individual patient data meta-analyses of NO donors, and BP lowering, for acute stroke (the latter through the ‘Blood pressure in Acute Stroke Collaboration’), and made available to participating countries and the ‘Virtual International Stroke Trials Archive’ 32.

Ultimately, a subset of the data will be made available over the web, as with the International Stroke Trial 33. Similarly, anonymized baseline and on-treatment neuroimaging data will be published 16.

## Supporting Information

Appendix S1 Statistical Analysis Plan (ENOS).
