# Supplementary material for: Statistical analysis plan for the ‘Efficacy of Nitric Oxide in Stroke’ (ENOS) trial
Source: Int J Stroke. 2014 Mar 3;9(3):372–4. doi: 10.1111/ijs.12235 (PMC4235425; doi:10.1111/ijs.12235)
Supplement: Appendix S1 — Statistical Analysis Plan (ENOS). [file ijs0009-0372-sd1.pdf]

STATISTICAL ANALYSIS PLAN

The Efficacy of Nitric Oxide in Stroke (ENOS) trial

Philip MW Bath, Aimee Houlton, Nikola Sprigg, Joanna Wardlaw, Stuart Pocock, on behalf of the ENOS Trialists

Author for correspondence:
Professor Philip M W Bath, MD FRCPath FRCP
Stroke Trials Unit, Division of Clinical Neurosciences, University of Nottingham, City Hospital campus, Hucknall Road, Nottingham NG5 1PB UK

Tel: +44 115 823 1765
Fax: +44 115 823 1767
E-mail: philip.bath@nottingham.ac.uk

CONTENTS

Appendix A Statistical Analysis Plan

1 Introduction
2 Primary research questions
3 Trial design
    3.1 Design
    3.2 Trial population
    3.3 Randomisation
        3.3.1 Stratification
        3.3.2 Minimisation
        3.3.3 Simple randomisation
    3.4 Minimising bias
    3.5 Ethics and regulatory approvals
4 Statistical analysis plan (SAP)
    4.1 Original SAP
    4.2 Revised SAP
    4.3 Outcomes and their analysis
        4.3.1 Analysis populations
            4.3.1.1 Intention-to-treat safety population
            4.3.1.2 Intention-to-treat efficacy population
            4.3.1.3 Per protocol population
            4.3.1.4 Analyses by population
        4.3.2 Missing data, and death
        4.3.3 Statistical assumptions
            4.3.3.1 Proportionality of odds
            4.3.3.2 Alpha spending
            4.3.3.3 mRS levels
    4.4 Primary outcome
        4.4.1 Primary outcome measure
        4.4.2 Primary outcome analysis
        4.4.3 Primary outcome subgroup analyses
            4.4.3.1 Primary publications
            4.4.3.2 Secondary publications
        4.4.4 Sensitivity analyses
    4.5 Secondary outcomes
        4.5.1 Events by day 7 (end of treatment)
        4.5.2 Hospital events
        4.5.3 Outcomes at day 90 (end of follow-up)
    4.6 Safety analyses – Serious Adverse Events
        4.6.1 SAEs by day 10
        4.6.2 SAEs by day 90
    4.7 Compliance
    4.8 Analysis methods
        4.8.1 Clinical variables
        4.8.2 Imaging variables
        4.8.3 Procedures

Appendix B Definitions

1 Definitions of events/outcomes
    1.1 Deep vein thrombosis (DVT), symptomatic, by/at day 7
    1.2 Disposition
    1.3 Extracranial haemorrhage, major

1.4 Feeding status
1.5 Headache, requiring treatment by day 7
1.6 Hypertension, symptomatic by day 7
1.7 Hypotension, symptomatic by day 7
1.8 Neurological deterioration, by day 7
1.9 Neurological deterioration, clinical by day 7
1.10 Pulmonary embolism, symptomatic by day 7
1.11 Recurrent stroke, symptomatic by day 7
1.12 Intracranial haemorrhage, symptomatic by day 7
1.13 Time at home
1.14 Venous thromboembolism, symptomatic by day 7
2 Other definitions and notes
2.1 Geographical region
2.2 Haemodynamic variables, calculated
2.3 Acute Stroke Unit
2.4 Stroke Rehabilitation Unit
2.5 Final diagnosis
2.6 Date of death
2.7 EQ-5D and Health Utility Status
2.8 Protocol violations
2.8.1 All analyses
2.8.2 GTN vs no GTN
2.8.3 Continue vs stop pre-stroke antihypertensive medication
2.9 CT/MR image adjudication
2.9.1 Infarct visibility
2.9.2 Lesion site
2.9.3 Sub-territory sites

Appendix C Tables and figures in primary publications

Authors
1 Table 1. Baseline characteristics
1.1 Both GTN/no GTN and Continue/Stop
1.2 Continue vs stop pre-stroke antihypertensive medications
2 Table 2. Compliance
2.1 Both GTN/no GTN and Continue/Stop
3 Table 3. Outcomes
3.1 Both GTN/no GTN and Continue/Stop
4 Table 4. Serious adverse events
4.1 Both GTN/no GTN and Continue/Stop
4.2 Continue vs stop pre-stroke antihypertensive medications
5. Table 5. Protocol violations
6.1 Both GTN/no GTN and Continue/Stop
6. Table A. Blood pressure
5.1 Both GTN/no GTN and Continue/Stop
7. Table B. mRS by treatment assignment
7.1 Both GTN/no GTN and Continue/Stop
8. Table C. Outcome by sub-groups
8.1 Both GTN/no GTN and Continue/Stop
9 Figure 1. Trial flow diagram
9.1 Both GTN/no GTN and Continue/Stop
10 Figure 2. Blood pressure profile
10.1 Both GTN/no GTN and Continue/Stop
11 Figure 3. Modified Rankin Scale distribution
11.1 Both GTN/no GTN and Continue/Stop

12 Figure 4. Primary outcome in pre-specified subgroups
 12.1 Both GTN/no GTN and Continue/Stop
13 Figure 5. Survival curve
 13.1 Both GTN/no GTN and Continue/Stop
14 Figure 6. Meta-analysis of relevant trials
 14.1 GTN/no GTN
 14.2 Continue/Stop

Appendix D Secondary publications

1 Published
2 Submitted
3 In preparation
4 Planned
 4.1 Treatment-related
 4.2 'Epidemiological'
 4.3 Systematic reviews/meta-analyses

Appendix E. Planned secondary publication: Baseline data

Appendix F. Planned secondary publication: Interventions in acute ischaemic stroke

Appendix G. Planned secondary publication: Interventions in acute spontaneous intracerebral haemorrhage

Appendix H. Planned secondary publication: Haemodynamic effects of GTN versus no GTN, and continue versus stop pre-stroke antihypertensive medications

Appendix I. Planned secondary publication: GTN, headache and outcome

Appendix J. Planned secondary publication: Effect of feeding status/dysphagia

Appendix K. Planned secondary publication: Comparison of spontaneous intracerebral haemorrhage between world regions

Appendix L. Comparison of patients who took versus did not take pre-stroke antihypertensive medications: baseline characteristics and outcomes.

Appendix M. Planned secondary publication: Temporal effects of GTN on functional outcome – a, individual patient data meta-analysis of GTN trials

Appendix N. Planned secondary publication: Continuing versus stopping pre-stroke antihypertensive medications – an individual patient data meta-analysis of trials

REFERENCES

KEYWORDS

acute stroke, antihypertensive drug, blood pressure, glyceryl trinitrate, randomised controlled trial, statistical analysis plan

APPENDIX A

1 INTRODUCTION

High blood pressure (BP) is present in 70% or more of patients with acute ischaemic stroke and spontaneous intracerebral haemorrhage (ICH).[1] Affected patients have a worse outcome, whether judged as early recurrence, death within a few weeks, or combined death and dependency after several months.[1-4] Lowering BP might therefore reduce these events and improve functional outcome providing that cerebral perfusion is not reduced in the presence of dysfunctional cerebral autoregulation. However, recent large trials have been inconclusive in their results.[5,6]

Nitric oxide (NO) donors are candidate treatments for acute stroke; NO is a cerebral and systemic vasodilator, modulates vascular and neuronal function, and inhibits apoptosis.[7] Preclinical studies of cerebral ischaemia have found that NO donors reduce stroke lesion size, and improve regional cerebral blood flow and functional outcome.[8] Five small clinical studies of NO donors have been performed, these involving a total of 208 patients with recent stroke. Intravenous sodium nitroprusside reduced BP without altering CBF, and exhibited antiplatelet effects (thereby precluding its use in spontaneous ICH).[9] Four pilot trials of transdermal glyceryl trinitrate (GTN) found that it lowered BP by approximately 8%; did not alter platelet function (and so could be given in ICH); did not alter middle cerebral artery blood flow velocity or regional cerebral blood flow; improved aortic vascular compliance; and could be given to patients with dysphagia.[10-13] No safety concerns were present in these studies, and in one small trial ultra-acute treatment with GTN was associated with an improved functional outcome.[13,14]

On the basis of this pre-clinical and clinical data showing feasibility, tolerability and apparent safety of GTN, and the potential for efficacy, the large 'Efficacy of Nitric Oxide in Stroke' (ENOS) trial was started and is ongoing. ENOS is assessing, in a partial factorial prospective randomised open blinded-outcome design, whether to lower BP with GTN (versus no GTN), and whether to continue (versus stop) pre-stroke antihypertensive therapy. The trial commenced in 2001 and its protocol was published in 2006.[15] Several non treatment-related and blinded analyses of the ENOS database have been published since the start of the trial.[16-20] The independent Data Monitoring Committee have assessed the trial every 6 months and on each occasion recommended that the trial should continue.

2 PRIMARY RESEARCH QUESTIONS

1. Does transdermal glyceryl trinitrate (GTN, 5 mg daily for 7 days), when given acutely after stroke, reduce death or dependency at 90 days, as compared to no GTN. This question applies to all randomised patients.

2. Does continuing pre-stroke antihypertensive medications reduce death or dependency at 90 days, as compared to stopping these medications for 7 days. This question applies to those randomised patients who were taking antihypertensive medications immediately prior to stroke onset (anticipated to be ~50% of the whole trial population).

3 TRIAL DESIGN

3.1 Design

ENOS is an international multicentre prospective randomised single-blind blinded-endpoint controlled partial-factorial trial with centralised randomisation (stratification and minimisation) to ensure allocation concealment and reduce bias.

3.2 Trial population

Previously independent adult patients who are conscious, have residual limb weakness, have systolic blood pressure between 140-220 mmHg, and are within 48 hours of stroke onset, are eligible for enrolment. The full inclusion-exclusion criteria are given in the protocol.[15]

3.3 Randomisation

All patients are randomised to GTN vs no GTN. Those patients who are taking antihypertensive medications immediately prior to their stroke are also randomised to continue or stop this. As a result, there are 6 treatment groups, with a working sample size totalling 3,800 patients (which exceeds the grant-funded 3,500 patients), and approximately 50% of patients on BP drugs before their stroke.

	Pre-stroke	BP drugs	No pre-stroke BP drugs	Size by row
	Continue	Stop		
GTN	475	475	950	1,900
No GTN	475	475	950	1,900
Size by column	950	950	1,900	3,800

The process of randomisation includes (1) stratification; (2) minimisation; and then (3) simple randomisation. Stratification and minimisation allow for improved matching at baseline, stratification allows variable categories to be treated as trials in their own right, minimisation increases statistical power,[21] and simple randomisation reduces predictability. The minimisation variables will be used for adjustment of the primary and secondary analyses.

3.3.1 Stratification

i. Immediate prior use of antihypertensive treatment.
ii. Stroke pathological type (ischaemic stroke [IS]/unknown, spontaneous intracerebral haemorrhage [ICH])
iii. Country

3.3.2 Minimisation

i. Age ($\leq$70 years, >70 years)
ii. Sex (female, male)
iii. History of hypertension (no, yes)
iv. History of previous stroke (no, yes)
v. Diabetes mellitus (no, yes)
vi. Current use of nitrate therapy (no, yes)
vii. Stroke severity (Scandinavian Stroke Scale,[22] SSS >30, $\leq$30)
viii. Total anterior circulation syndrome (no, yes) [23]
ix. Systolic BP ($\leq$160, >160 mmHg)
x. Treated with rt-PA prior to randomisation (yes, no)
xi. Feeding status (other, iv fluids/nothing)
xii. Time to randomisation ($\geq$24 hours, <24 hours)

3.3.3 Simple randomisation

On 5% of patients at time of minimisation.

3.4 Minimising bias

Multiple measures are taken to minimise bias: central data registration with real-time on-line validation; concealment of allocation; patient blinding to GTN (gauze dressing over GTN patch or equivalent area of skin); blinded central telephone assessment of 3 month outcome by staff at National Coordinating Centres; assessment of patient recall of treatment;[11] blinded adjudication of CT scans/SAEs; exclusion of patients enrolled in other trials; analysis by intention-to-treat; and adjustment for minimisation factors and non-randomised treatment (alteplase).

3.5 Ethics and regulatory approvals

ENOS is run according to the principles of the Declaration of Helsinki and 'Good Clinical Practice'. The trial was approved by national competent authorities (where applicable), e.g. European Medicines Agency (EudraCT number 2004-003870-27, date 1/10/2004), UK Medicines & Healthcare products Regulatory Authority (formerly Medicines Control Agency, reference MF8000/10820, date 16/2/2001); national and local research ethics committees (REC), e.g. UK Trent REC (reference MREC/01/4/046, date 3/9/2001), Scottish 1 REC (reference MREC/02/0/121, date 7/1/2003); and adopted by the Australian, Canadian, and UK NIHR Stroke Research Networks (date 25/4/2006). Trial funding from BUPA Foundation, and the Medical Research Council (grant: G0501797, awarded 26/4/2006) followed external peer review.

4 STATISTICAL ANALYSIS PLAN (SAP)

4.1 Original SAP

The trial was originally designed to recruit 5,000 patients so as to detect an absolute risk reduction in the binary outcome of death or dependence (modified Rankin Scale, mRS>2) of 5% from 50% in the control group to 45% in the GTN group, with power 90%, significance 5%, and allowance for losses to follow-up. The original planned method of analysis, as published in the protocol paper,[15] was to compare the proportion of participants who were dead or dependent at 90 days between the treatment groups.

4.2 Revised SAP

Recent research has shown that binary analysis of the 7-level mRS is sub-optimal and that statistical power is increased by using all the data at each level by comparing differences in distribution across the whole scale between the treatment groups.[24] This approach is now recommended by the European Stroke Organisation.[25] A further, and additional, increase in statistical power is achieved by incorporating key prognostic baseline variables as covariates.[26] Other groups have presented similar findings and used this approach.[27-29]

Hence, the primary analysis of the mRS in ENOS will utilise the shift approach, as analysed using ordinal logistic regression, with adjustment for covariates, with comparison between treatment groups (GTN/no GTN; continue/stop pre-stroke BP medications). The overall proposal to change the method of analysis of the primary outcome from binary to ordinal was first presented to, and agreed by, the Trial Steering Committee in January 2008, and confirmed in 2009. An early draft version of this SAP, highlighting this change, was posted on the trial website in April 2009.

This change to the design of ENOS was made without knowledge of any interim analysis that split patients by treatment group. The statistician who prepares analyses for the independent Data Monitoring Committee (DMC), and the DMC themselves, were not

involved in the writing of this statistical analysis plan (SAP), and have not seen or commented on it.

4.3 Outcomes and their analysis

All outcomes will be assessed by:
- GTN versus no GTN
- Continue versus temporarily stop pre-stroke antihypertensive medication

4.3.1 Analysis populations
The following populations of patients are defined for analyses and take account that the trial is single-blind in design.

4.3.1.1 Intention-to-treat safety population
All randomised participants with vital status recorded.

4.3.1.2 Intention-to-treat efficacy population
All randomised participants with the primary outcome recorded.

4.3.1.3 Per protocol population
All randomised participants with the primary outcome recorded and who did not have a relevant protocol violation as defined in ***Appendix B.2.8***. A key criterion is that an included patient must have received at least 4 days of treatment (or died before this).

4.3.1.4 Analyses by population
All efficacy analyses will be performed on the intention to treat population. A separate 'per protocol' analysis will be performed on the primary and key secondary outcomes. Safety analyses will be performed on the safety population.

4.3.2 Missing data, and death

Missing data will not be imputed. Participants who die will be assigned discrete values for outcome measures with a value worse than any living value (as is standard for mRS, BI). This avoids giving death the same value as the worst possible outcome when alive (best to worst), and ensures that patients who die are included in all analyses. The EQ-5D Health Utility State (HUS) gives death a score of 0.
- Modified Rankin Scale (mRS), 0 to 5 with death = 6
- Barthel Index (BI), 100 to 0 with death = -5
- Scandinavian Stroke Scale (SSS), 58 to 0 with death = -1
- EQ-5D HUS, 1 to -0.594 with death = 0
- EQ-VAS (visual analogue scale), 100 to 0 with death = -1
- Zung Depression Scale (ZDS), 25 to 100 with death = 102.5
- Telephone Interview for Cognitive Status (TICS), 39 to 0 with death = -1
- Verbal fluency (animal naming), ∞ to 0, with death = -1
- Telephone Mini Mental Status Exam (t-MMSE), 18 to 0, with death = -1

Patients who are still in hospital at day 90 will have a length of stay assigned at 110 days. Similarly, death will be censored at day 110.

4.3.3 Statistical assumptions

4.3.3.1 Proportionality of odds
The treatment effects on ordered categorical data will be compared using ordinal logistic regression (OLR) with adjustment for the prognostic covariates as listed in 3.3.2, plus use of alteplase. OLR assumes proportionality of odds and this will be tested using a

likelihood ratio test. If the proportional odds assumption is not met, OLR will still be used and the lack of proportionality of odds highlighted. In this circumstance, the odds ratio may remain relevant (and probability value accurate) providing the treatment effect is relatively consistent across the spread of mRS scores.

4.3.3.2 Alpha spending
The Data Monitoring Committee performs safety assessments using the '3 standard deviation' approach; hence, no significant spending of alpha has occurred during the trial. All analyses will be two-tailed and a p-value of <0.05 will denote statistical significance; 95% confidence intervals will be provided. Adjustment for multiple comparisons will not be performed but all contrasts will be declared.

The primary and secondary outcomes follow here. Although not all the secondary outcomes will be presented in the two primary papers (GTN vs no GTN, continue vs stop pre-stroke antihypertensive medication), they will be used in secondary papers and are given here for completeness.

4.3.3.3 mRS levels
Although some previous studies (e.g. SAINT-I/II, ECASS-3 [29-31]) combined death with severe disability (i.e. mRS categories 5 and 6 were combined), these mRS categories will be analysed separately in ENOS since blood pressure treatment may influence both mortality and disability.

4.4 Primary outcome

4.4.1 Primary outcome measure
The seven-level ordered categorical modified Rankin Scale (mRS):[22,32]

0 No symptoms at all.
1 No significant disability, despite symptoms; able to carry out all usual duties and activities.
2 Slight disability; unable to carry out all previous activities but able to look after own affairs without assistance.
3 Moderate disability; requiring some help, but able to walk without assistance.
4 Moderately severe disability; unable to walk without assistance and unable to attend to own bodily needs without assistance.
5 Severe disability; bedridden, incontinent and requiring constant nursing care and attention.
6 Dead.

4.4.2 Primary outcome analysis
All 7 levels of the mRS will be used with analysis by OLR with adjustment for baseline covariates:[24-26]

- Age; sex (female, male); pre-morbid mRS; history of previous stroke (no, yes); diabetes mellitus (no, yes); current use of nitrate therapy (no, yes); stroke severity (Scandinavian Stroke Scale, SSS); total anterior circulation syndrome (no, yes); stroke pathological type (non, IS, ICH); systolic BP (SBP); treated with rt-PA (yes, no); feeding status (oral, non oral); time onset to randomisation.
- The comparison of GTN vs no GTN will also include the covariate for Continue vs stop pre-stroke antihypertensive medication.
- The comparison of Continue vs stop pre-stroke antihypertensive medication will also include the covariate for GTN vs no GTN.

Covariate adjustment with ordered categorical (mRS) or continuous (age, SSS, SBP, time) variables will use the original, not dichotomised, data.

4.4.3 Primary outcome subgroup analyses

The effect of the two interventions (GTN versus no GTN; continue versus temporarily stop pre-stroke antihypertensive medication) on the primary outcome will be performed within the following pre-specified subgroups of participants. Subgroup analyses will be performed on mRS using ordinal logistic regression. An interaction test between each subgroup and treatment will be performed.

4.4.3.1 Primary publications

a) Age - ≤70, >70 years
b) Sex – female, male
c) History of prior stroke – no, yes
d) Stroke severity (/58) – SSS >40, 30-40, <30
e) Oxfordshire Community Stroke Project Classification [23] – LACS, POCS, PACS TACS
f) Stroke pathological type – ischaemic stroke, unknown, ICH
g) Mean blood pressure (mmHg) – SBP ≤160, 161-180, 181-200, >200
h) Atrial fibrillation/flutter – absent, present
i) Non-oral feeding (surrogate for dysphagia) – absent, present
j) Carotid stenosis (ipsilateral %) – 0-49, 50-69, 70-99, 100 (as determined locally)
k) Time to randomisation (hours) - ≤6.0, 6.1-12.0, 12.1-24.0, 24.1-36, >36
l) Treatment with alteplase – present, absent

Additionally, for GTN versus no GTN

a) History of recent nitrate exposure – no, yes
b) Pre-stroke antihypertensive medication randomisation – not relevant, continue, stop

Additionally, for continue versus stop pre-stroke antihypertensive medication:

a) Pre-stroke antihypertensive class – angiotensin-converting enzyme inhibitor, angiotensin-II receptor antagonist, renin inhibitor, β-blocker, calcium-channel blocker, diuretic, α-blocker, centrally acting agent, other (participants may appear in more than one subgroup)
b) Number of classes of antihypertensive drugs pre-stroke – 1, 2, 3, 4, 5, >5
c) GTN randomisation – GTN, no GTN

4.4.3.2 Secondary publications

In secondary papers, the effect of treatment on mRS will also be assessed in additional sub-groups:

a) Geographical region (section B.2.1)
b) TOAST criteria (small vessel, large vessel, cardioembolism, dual pathology, other)
c) Carotid stenosis (>=50%) – none, contralateral, ipsilateral, bilateral
d) Old lesion on baseline neuroimaging – no, yes
e) Lesion size – 1, 2, 3, 4 (Appendix B, section 2.9.2)
f) Mass effect – 0,1 vs 2-4 vs 5,6 (Appendix B, section 2.9.3)

4.4.4 Sensitivity analyses

Several sensitivity analyses will be performed as a reference for the primary analysis. The method of analysis is given: BLR: binary logistic regression; OLR: ordinal logistic regression

a) mRS, unadjusted	OLR
b) mRS, with additional adjustment for Geographical region	OLR
c) Dead or dependent (mRS 3-6, adjusted)	BLR
d) Dead or dependent (mRS 3-6, unadjusted)	BLR
e) Global outcome, mRS>1 and BI <95, adjusted	Wald test [33,34]

Comparison (d) is the original trial primary outcome prior to switching to an ordinal analysis.

4.5 Secondary outcomes

Secondary outcomes are listed by their timing. Analysis methods will include: MLR: multiple linear regression; analyses will be adjusted as in section 4.4.2 and also performed unadjusted for completeness.

4.5.1 Events by day 7 (end of treatment)

a) Death	BLR
b) Symptomatic intracranial haemorrhage	BLR
c) Major extracranial haemorrhage	BLR
d) Symptomatic recurrent stroke	BLR
e) Impairment (SSS)	MLR
f) Impairment (calculated NIHSS [35])	MLR
g) Clinical neurological deterioration	BLR
h) Neurological deterioration [22]	BLR
i) Headache that required treatment	BLR
j) Symptomatic hypotension that required treatment	BLR
k) Symptomatic hypertension that required treatment	BLR
l) Symptomatic venous thromboembolism	BLR

4.5.2 Hospital events (collected at discharge or on death)

a) Length of stay in hospital	MLR
b) Discharge disposition (death/institution/home)	OLR

4.5.3 Outcome at 90 days (end of follow-up)

This are determined centrally by a telephone call between the patient (or carer) and an assessor blinded to treatment and earlier clinical information:

a) Death (censored by day 110)	BLR
b) Time to death (censored at 110 days)	Cox regression (Kaplan Meier curve)
c) Barthel Index (BI)	MLR
d) Dead or disabled (BI <60)	BLR
e) Quality of life (HUS derived from EQ-5D)	MLR
f) Quality of life (EQ-5D VAS)	MLR
g) t-MMSE	MLR
h) TICS-M	MLR
i) Verbal fluency	MLR
j) Zung Depression Scale (ZDS, mood)	MLR

4.6 Safety analyses – Serious Adverse Events

Safety analyses are listed together with their preferred method of analysis. All SAEs reported by the investigator are adjudicated by a member of the independent adjudicator panel who is blinded to treatment. The adjudicated SAE categorisation will be presented for all analyses. The number of participants with a SAE rather than the number of SAE will be analysed. Comparisons will be performed on all patients.

4.6.1 SAEs by day 10 (7 days of treatment + 3 days of washout)

a) Serious adverse event, number of patients	BLR

4.6.2 SAEs by day 90 (end of follow-up)

a) Serious adverse event, number of patients	BLR

Serious adverse events by 90 days will be tabulated as the number of participants with an event in each treatment group, and the number of events in each treatment group,

by the following subgroups. BLR will be used to identify any differences between treatment groups.

a) System organ class
b) Event category within system organ class
c) Time of onset relative to treatment (before, during, after)
d) Median time to event by event category
e) Severity (mild, moderate, severe)
f) Relationship to study drug (definitely not, unlikely, possibly, probably, definitely)
g) Fatal, non-fatal
h) Fatal by event category

Common serious adverse events that will be reported are listed in Appendix C table 4.

4.7 Compliance

Compliance with allocated treatment will be tabulated. Patients receiving at least the first 4 doses of treatment are considered to have received full treatment. The following data will be presented separately for GTN vs. no GTN, and continue vs. temporarily stop pre-stroke antihypertensive medications

a) Number (%) of participants who received all 7 days of allocated treatment
b) Number (%) of participants who received at least the first 4 days of allocated treatment
c) Number (%) of participants who received at least the first allocated treatment
d) Number (%) of participants who did not receive any of the allocated treatment

In those participants who did not receive all the first 4 days of allocated treatment, the reason for non-compliance will be given:

a) Discharged before day 4
b) Physician withdrew participant
c) Adverse event, unacceptable
    a. Headache
d) Serious adverse event
e) Consent withdrawn by participant or relative
f) Death
g) Other reason

Reasons for non-compliance	All	GTN	No GTN
Reasons for non-compliance	All	Continue	Stop
Participants randomised			
Compliance			
Received all 7 days of treatment			
Received first 4 days of treatment			
Received first treatment			
Did not receive any randomised treatment			
Non-compliance by day 4 (%)			
Discharged			
Physician withdrew participant			
Adverse event, unacceptable			
Headache			
Serious adverse event			
Consent withdrawal by participant or relative			
Death			
Other			

Note: Patients discharged before day 7 were given their remaining part of their course of GTN to complete at home.

4.8 Analysis methods

Data variables will, in the main, be analysed using binary logistic regression (BLR), Cox logistic regression (CLR); ordinal logistic regression (OLR) or multiple logistic regression (MLR), thereby allowing adjustment for baseline measures and other covariates (as listed partially in section 4.4.3).

4.8.1 Clinical variables

- BLR: Death; Death or deterioration (day 7); Recurrence (day 7); Symptomatic intracranial bleed (sICH); Discharged to or at Home
- CLR: Death
- OLR: Modified Rankin Scale (mRS) [/6];
- MLR: Scandinavian Stroke Scale (SSS, /58); Length of stay in hospital; Barthel Index (BI, /100); telephone Mini-Mental State Examination (tMMSE); Telephone Interview of Cognition Scale (TICS, /39); Animal naming (/∞); Zung Depression Scale (ZDS, /100); Health Utility State (HUS, /1); EuroQoL Visual Analogue Scale (EQ-VAS, /100)

4.8.2 Imaging variables

- BLR: Loss of grey-white definition; Loss of basal ganglia outline; Hypodensity; Mass effect; ICH mean radial expansion > 1mm; Second lesion; Hyperdense artery
- OLR: Lesion site; Lesion size: MCA, lacunar, cerebellum or brainstem; Mass effect, degree [/6]; Haemorrhagic Transformation of Infarct; Hypoattenuation; Mass effect score (greatest of Uncal> shift> ventricular> sulcal); Middle Cerebral Artery 1/3 (if MCA, >1/3, <1/3, no lesion); ASPECTS score; ICH diameter, max – adjudicated; ICH shape [/5]; ICH density [/5]; IVH Graeb [/12];[36] IVH modified Graeb [/32];[37]
- MLR: Volume (ABC/2), absolute (ml); Volume (ABC/2), relative (%); Diameter, max (cm); Haematoma shape index; Haematoma mean density; Haematoma density index; IVH volume

4.8.3 Procedures

The two primary papers will be analysed using two separate sets of statistical programmes, one written specifically for the purpose and the other based on the Data Monitoring Committee report. A further analysis of a sub-set of the main results will be performed using a third statistical programme. This will allow the results to be cross-checked.

To maximise recruitment, maximise time for analysis and interpretation of the results, and allow presentation of the final results at the European Stroke Conference in early May 2014, a soft-lock and preliminary analysis will be performed once the last patient has had their final outcome recorded in early January 2014. Following final data cleaning on the last patients to be recruited, a hard-lock will be performed in late January/early February and the results of this considered by the Trial Steering Committee in mid February 2014. The preliminary interpretation will be performed after soft-lock by PMB, NS, JW and SP; they will not be involved in resolving any final queries to maintain the integrity and blinding of the final database. The approach of soft-lock then hard-lock is a standard approach in large trials and does allow more time to be spent on considering the results of a trial, their interpretation and presentation for publication.

APPENDIX B. DEFINITIONS

1 DEFINITIONS OF EVENTS/OUTCOMES

1.1 Deep vein thrombosis (DVT), symptomatic, by/at day 7
The clinical suspicion of DVT will need confirmation by either venography or ultrasound examination.

1.2 Disposition
Disposition at 90 days and discharge disposition is categorised as death, institution or home.
- Institution refers to warden controlled, residential home, care home, nursing home, still an inpatient, or readmitted to hospital.
- Home refers to home alone, home with spouse/carer and carer's home.

1.3 Extracranial haemorrhage, major
Clinically overt bleeding associated with one or more of:
- Transfusion of > 2 red cell units of blood
- A fall in haemoglobin of 20 g/l (=2 g/dl, = 1.24 mmol/l)
- Bleeding into retroperitoneum, intraocular space or major joint
- Bleeding leading to permanent cessation of trial interventions

1.4 Feeding status
Feeding status will be defined as:
- Oral: normal diet, soft diet
- Non-oral: nasogastric tube fed, PEG-tube fed, iv/sc fluids, no feeding/fluids

1.5 Headache, requiring treatment by day 7
A headache occurring during GTN/no GTN treatment that necessitates intervention, including withdrawing GTN/no GTN treatment.

1.6 Hypertension, symptomatic by day 7
A rise in blood pressure necessitating intervention to reduce it.

1.7 Hypotension, symptomatic by day 7
A symptomatic fall in blood pressure of > 20% as compared with baseline necessitating intervention with intravenous colloid or crystalloid (saline).

1.8 Neurological deterioration, by day 7
A reduction in SSS of >4 points, or decrease in consciousness level by >2 points, as compared with baseline.[22]

1.9 Neurological deterioration, clinical by day 7
Neurological deterioration determined clinically.

1.10 Pulmonary embolism (PE), symptomatic by day 7
The clinical suspicion of PE will need confirmation by either high-probability ventilation-perfusion lung scintigraphy, pulmonary angiography, inconclusive V/Q scan and DVT, or lead to death.

1.11 Recurrent stroke, symptomatic, by/at day 7
Classified as haemorrhagic or ischaemic (if documented by CT/MR scan or autopsy), or of unknown type. The time from stroke onset and side will be noted. (This definition deliberately does not attempt to differentiate true recurrence from extension of the presenting lesion since this is clinically and radiologically difficult unless recurrence

occurs in a new arterial territory.)

1.12 Intracranial haemorrhage, symptomatic by day 7
Neurological deterioration or death, associated with intracranial haemorrhage found on CT/MR scan or autopsy.

1.13 Time at home
Calculated as time from date of discharge to day 90 or death if earlier; those who die in hospital or are discharged to a non-home setting are given a score of zero. Readmission to hospital is not counted in this time.

1.14 Venous thromboembolism, symptomatic by day 7
Symptomatic DVT and/or symptomatic pulmonary embolism

2 OTHER DEFINITIONS AND NOTES

2.1 Geographic region
This is categorised into Africa, America north, Asia east, Asia south, Asia south-east, Australasia, British Isles, and Europe. Each geographical region is defined as the following countries, each of which have randomised at least one participant into the ENOS trial:

a) Africa: Egypt
b) America, north: Canada
c) Asia, east: China, Hong Kong
d) Asia, south: India, Sri Lanka
e) Asia, south-east: Malaysia, Philippines, Singapore
f) Australasia: Australia, New Zealand
g) British Isles: Eire, UK
h) Europe: Denmark, Georgia, Greece, Italy, Norway, Poland, Romania, Spain, Sweden

2.2 Haemodynamic variables, calculated
These are calculated from SBP, diastolic blood pressure (DBP) and HR measurements at baseline (day 0) and on days 1-6. Mean, peak, and standard deviation (SD), of SBP/DBP/HR are calculated from available measurements at that time point.

Coefficient of Variation, CV=SD/mean.

a) SBP, mean
b) SBP, peak
c) SBP, intra-visit variability = SD of SBP separately at each time point
d) SBP, CV
e) DBP, mean
f) DBP, peak
g) DBP, intra-visit variability = SD of DBP separately at each time point
h) DBP, CV
i) HR, mean
j) HR, peak
k) HR, intra-visit variability = SD of HR separately at each time point
l) HR, CV
m) Mean arterial pressure (MAP) = mean DBP + (mean SBP / 3)
n) Mean pressure (MP) = (mean DBP + mean SBP) / 2
o) Pulse pressure (PP) = mean SBP – mean DBP
p) Pulse pressure index (PPI) = PP / MAP

q) Rate-pressure product (RPP) = mean SBP x mean HR
r) Mean SBP across days 1-4
s) Inter-visit Variability (SBP-inter SD) = SD of SBP across days 1-4
t) CV SBP across days 1-4
u) SBP peak across days 1-4

2.3 Acute Stroke Unit (ASU)

A high-dependency nursing unit (or area) caring only/mainly for patients with acute stroke and providing close monitoring of neurological and vascular signs.

2.4 Stroke Rehabilitation Unit (SRU)

A dedicated rehabilitation unit (or area) caring only/mainly for patients with recent stroke and providing multi-disciplinary therapy (e.g. physiotherapy, occupational therapy, speech & language therapy).

2.5 Final diagnosis: ischaemic stroke, ICH, unknown type of stroke, non-stroke

The final diagnosis for the qualifying event will be determined using information from the recruiting site based on the clinical presentation, history of recovery and interpretation of the brain scan. The resulting diagnosis will be compared with that from central adjudication of the baseline brain scan. If there is a discrepancy between site and adjudication, the site will be contacted to clarify the diagnosis and the scan reviewed centrally by JMW; ultimately the site's final diagnosis will be used if agreement cannot be obtained.

Final diagnosis will comprise one of the following: ischaemic stroke (which includes a normal CT brain scan), ICH, stroke of unknown pathological type, or non-stroke (e.g. tumour, abscess, subdural bleed).

Final diagnosis will be determined using the following source(s):

1. Hospital event form (completed at discharge or death: final diagnosis question). If this is unavailable use 2.
2. Day 7 form (baseline CT/MRI scan result question). If this is unavailable use 3.
3. Baseline form – as used for randomisation (baseline CT/MRI scan result question). If this is unavailable use 4.
4. If the diagnosis of stroke is not known according to the data sources above (e.g. due to early death and no autopsy), then the diagnosis will be assigned as 'Stroke, type unknown'.

2.6 Date of death

Dates of death have multiple sources and these are ranked for determining the date (in case a particular source is missing the information):

1. MRIS/ONS feedback, if UK patient
2. Serious adverse event form
3. Hospital event form
4. Day 7 form

If there is a discrepancy in the recorded date then the site are approached to confirm the correct date.

2.7 EQ-5D and Health Utility States (HUS)

EQ-5D is a measure of health status and is composed of the EQ-5D descriptive system and EQ visual analogue scale (EQ VAS). The EQ VAS is a self-rated scale from 0 (worst imaginable health state) to 100 (best imaginable health state). The EQ-5D descriptive system has 5 dimensions: mobility, self-care, usual activities, pain/discomfort and anxiety/depression. Each dimension has 3 levels; no problems (level 1), some problems (level 2), severe problems (level 3).[38]

There are 243 possible health states. Each health state is referred to by a 5 digit code, e.g., 11111. The EQ-5D health states can be converted into a single summary index ranging from 1.00, i.e. full health (11111), to -0.594 (33333, worst possible state using the UK version of the time trade-off algorithm), with death assigned to 0.[38] The conversion is made by applying the formula in Table 5, which deducts weights applied to each dimension from the starting value of 1. The UK version of the EQ-5D index algorithm using the time trade off value set will be used for all patients.[19]

EQ-5D index scoring algorithm (Time trade-off: UK version)

	Weight
Full health (11111)	1
At least one 2 or 3	-0.081
At least one 3	-0.269
Mobility	
Score 2	-0.069
Score 3	-0.314
Self-care	
Score 2	-0.104
Score 3	-0.214
Usual activities	
Score 2	-0.036
Score 3	-0.094
Pain/discomfort	
Score 2	-0.123
Score 3	-0.386
Anxiety/depression	
Score 2	-0.071
Score 3	-0.236

For example, a health state of 32211 will convert to 0.196 (=1-0.81-0.269-0.314-0.104-0.071).

2.8 Protocol violations
For per protocol analyses, patients who have one or more of the following protocol violations will be excluded from the per protocol population:

2.8.1 All analyses
- Failure to obtain consent or assent
- Antihypertensive medication received post-stroke and pre-randomisation
- Age<18 years
- Randomised >48 hours
- No limb weakness at any point in presentation
- Glasgow Coma Scale (GCS) <8 at baseline
- Systolic BP <140 mmHg or >220 mmHg at baseline
- Final diagnosis non-stroke
- Final diagnosis unknown because no neuroimaging or autopsy

2.8.2 GTN vs no GTN
- Failure to stop pre-stroke nitrates
- No GTN received for first 4 days if randomised to GTN
- GTN received in first 4 days if randomised to no GTN

2.8.3 Continue vs stop pre-stroke antihypertensive medication
- Failure to identify pre-stroke antihypertensive medication

- Antihypertensives received during first 4 days if randomised to stop
- Antihypertensives not received during first 4 days if randomised to continue

2.9 CT/MR image adjudication

All patients should have a diagnostic CT or MRI scan, ideally before randomisation or at least within one week of randomisation. A second CT or MRI scan is performed at the end of treatment (7 ± 2 days, or earlier if clinically warranted) to allow the effect of treatment on stroke lesion development to be assessed; principal investigators can opt in or out of the second scan. Both baseline and second CT/MRI scans are assessed by an independent adjudicator blinded to treatment assignment. Lesion size is assessed by an independent assessor, i.e. not by the investigator.

2.9.1 Infarct visibility (as per IST-3 scheme ©JMW 2012 [39])

Infarct size is coded (small=1 through to large=4) using adjudicated data for both baseline and, where done, day 7 CT/MR scans.

**2.9.2 Site**	**Condensed code**
M† =MCA = any lesion in the MCA territory	see 2.13.10.2 below
AS =Infarct of up to half of ACA territory	1
AL =Infarct of more than half of ACA territory	2
PS =Infarct of up to half of PCA territory	1
PL =Infarct of more than half of PCA territory	3
MAS=M+AS†	3 if MCA 1 or 2; 4 if MCA 3 or 4
MAL=M+AL†	3 if MCA 1 or 2; 4 if MCA 3 or 4
MPS=M+PS†	3 if MCA 1 or 2; 4 if MCA 3 or 4
MPL=M+PL†	3 if MCA 1 or 2; 4 if MCA 3 or 4
MAP=Infarct of whole MCA, ACA and PCA territories	4
L† =Lacunar	1
B† =Borderzone	1
C† =Cerebellum	see b below
S† =Brainstem	see b below
CS†=Cerebellum and brainstem	3

† code sub-territory sites as in 2.13.10.2

2.9.3 Sub-territory sites

MCA sub-territory codes	
1=small cortical infarct	1
2=basal ganglia infarct (>2x2x2cm)	2
3=infarct of white matter lateral to lateral ventricle (>2x2x2cm)	2
4=infarct of anterior half of peripheral MCA territory	2
5=infarct of posterior half of peripheral MCA territory	3
6=infarct of whole of peripheral MCA territory	3
7=6+infarct of lateral part of basal ganglia	4
8=infarct of whole of MCA territory	4

Lacunar/Borderzone sub-territory codes	all 1
9=lacune in internal capsule/lentiform	
10=lacune in internal border zone	
11=lacune in centrum semiovale	
12=lacune in thalamus	
13=lacune in brainstem, including pons (not shown)	
14=anterior (mainly) border zone	
15=posterior (mainly) border zone	

Cerebellum sub-territory codes

16=small cortical (not shown)	1
17=<1/2 hemisphere (medium) (not shown)	2
18=>1/2 hemisphere (not shown)	3

Brainstem sub-territory codes

19=small, i.e.<1/2 medulla (not shown)	1
20=extensive, i.e. pons + medulla (not shown)	2

Degree of mass effect codes
0=no swelling
1=effacement of sulci overlying stroke
2=1+minor effacement of adjacent lateral ventricle
3=1+complete effacement of adjacent lateral ventricle
4=1+effacement of lateral and third ventricle
5=4+shift of the midline away from side of ventricle
6=5+effacement of basal cisterns

2.9.4 Infarcts

Loss of grey/white matter cortex definition	yes/no
Loss of basal ganglia outline	yes/no
Hypodensity present	yes/no
Mass effect	yes/no, if yes each yes/no

- Succal effacement
- Ventricular effacement
- Midline shift
- Uncal herniation

1/3 MCA territory	yes/no
ASPECT score, for each region	yes/no
Second discrete lesion	yes/no
Hyperdense artery	yes/no, if yes then site

2.9.5 Haemorrhage

By presence, importance and size (<3, 3-5, 5-8, >8 cm):

- Petechial
- Haemorrhagic transformation of infarct
- Parenchymal haematoma (no infarct)
- Parenchymal haematoma (remote from infarct)
- Subdural haematoma
- Subarachnoid haemorrhage
- Extradural haemorrhage

2.9.6 Other features

Reduction in brain tissue volume: none, moderate severe:

- Central
- Cortical

Periventricular lucencies: none, restricted to region adjoining ventricles, from ventricle to cortex

- Anterior white matter
- Posterior white matter

Old vascular lesions: each yes/no

- Cortical infarct(s)
- Striatocapsular infarct(s)
- Borderzone infarct(s)
- Lacunar infarct(s)
- Brainstem/cerebellar infarct(s)

Non-stroke lesions, each yes/no

- Cerebral tumour
- Encephalitis
- Cerebral abscess
- Other, e.g. contusion

APPENDIX C. TABLES AND FIGURES IN PRIMARY PUBLICATIONS

Authors

ENOS Trial Investigators

Format

The primary publications for the GTN vs no GTN, and continue vs stop pre-stroke antihypertensive medications will share the same format for tables and figures as highlighted below. Any additions to, or differences in, one or other publication are given separately.

1 Table 1. Baseline characteristics

1.1 Both GTN/no GTN and Continue/Stop publications

Table 1. Baseline characteristics of patients enrolled into the ENOS trial. Data are number (%), median [interquartile range] or mean (standard deviation).

	All patients	GTN	No GTN
	All patients	Continue	Stop
Number of patients			
Age (years) †			
Sex, male (%) †			
Geographical region ‡			
Africa			
America, north			
Asia, east			
Asia, south			
Asia, south-east			
Australasia			
British Isles			
Europe, mainland			
Pre-stroke mRS [/6]			
Medical history (%)			
Treated hypertension ‡			
Atrial fibrillation, current/previous			
Stroke			
Ischaemic heart disease			
Peripheral arterial disease			
Smoking, current (%)			
Qualifying event (%) †			
Ischaemic stroke			
Spontaneous intracerebral haemorrhage			
Stroke type unknown			
Non-stroke			
Side of lesion, right (%)			
Scandinavian Stroke Scale (/58) †			
NIHSS (/42), calculated [40]			
Glasgow Coma Scale [/15]			
OCSP classification (%)			
Total anterior †			
Partial anterior			
Lacunar			
Posterior			
TOAST classification (%) Ω∫			

Cardioembolic
Large vessel
Small vessel
Mixed
Other
Haemodynamics
Systolic blood pressure (mmHg) †
Diastolic blood pressure (mmHg)
Heart rate, mean (bpm)
Oral feeding (%)
Time onset to randomisation (hours) †
Thrombolysis †

† Minimisation variable; ‡ Stratification variable; Ω Ischaemic patients only; Δ Protocol violation

bpm: beats per minute; NIHSS: National Institutes of Health Stroke Scale; mRS: modified Rankin Scale; OCSP: Oxford Community Stroke Project TOAST: Trial of ORG 10172 in Acute Stroke Treatment

1.2 Continue vs stop pre-stroke antihypertensive medications

The following variables will also be given in table 1 of this primary paper.

	All patients	Continue	Stop
Number of patients			
…			
Medications (%)			
Blood pressure lowering			
Angiotensin converting enzyme inhibitor			
Angiotensin receptor antagonist			
Renin inhibitor			
ß-receptor antagonist			
Calcium channel blocker			
Diuretic			
α-receptor antagonist			
Centrally acting			
Other			
Blood pressure, number taken, median			
0 (%) Δ			
1 (%)			
2 (%)			
3 (%)			
4 (%)			
5 (%)			
6 (%)			
…			

2. Table 2. Compliance

2.1 Both GTN/no GTN and Continue/Stop publications

Table 2. Compliance and reasons for non-compliance from randomised treatment. Patients receiving at least first 4 doses are considered to have had full treatment. Data are number (%) Comparison of total withdrawals by binary logistic regression with 95% confidence intervals.

Reasons for non-compliance	All	GTN	No GTN
Reasons for non-compliance	All	Continue	Stop
Participants randomised			
Compliance			
Received all 7 days of treatment			
Received first 4 days of treatment			
Received first treatment			
Did not receive any randomised treatment			
Non-compliance by day 4 (%)			
Discharged			
Physician withdrew participant			
Adverse event, unacceptable			
Headache			
Serious adverse event			
Consent withdrawal by participant or relative			
Death			
Other			

Odds ratio= , 95% confidence interval = - , 2p= .
Note: Patients who were randomised to GTN and were discharged before day 7 were given their remaining GTN patches to take in the community

3 Table 3. Outcomes

3.1 Both GTN/no GTN and Continue/Stop publications

Table 3. Primary and secondary outcomes at 7 and 90 days. Data are number (%), median [interquartile quartile range] or mean (standard deviation). Comparison by binary logistic regression, ordinal logistic regression, or multiple linear regression, shown as odds ratio (OR) median [interquartile range] or mean difference (MD), with 95% confidence intervals. Analyses are adjusted unless stated.

Outcome	GTN	No GTN	OR/MD (95% CI), adjusted	2p
Outcome	Continue	Stop	OR/MD (95% CI), adjusted	2p
Primary outcome				
Modified Rankin Scale (/6)			OR	
Sensitivity analyses				
mRS (/6), adjusted plus geographical region				
mRS (/6), unadjusted				
mRS>2				
mRS>2, unadjusted				
Global outcome	-	-		
Secondary outcomes				
Day 7 (or discharge)				
Death, by cause (%)				
Stroke progression			-	
Recurrent stroke			-	
Intra-cranial bleeding			-	
Extra-cranial bleeding			-	
Myocardial infarction			-	
Other cardiovascular cause			-	
Pulmonary embolism			-	
Pneumonia			-	
Other sudden death			-	
Other cause			-	
Other infection			-	
Symptomatic intracranial haemorrhage (%)				
Major extracranial haemorrhage (%)				
Symptomatic recurrent stroke, all (%)				
Ischaemic			-	
Haemorrhage			-	
Unknown			-	
Scandinavian Stroke Scale (SSS, /58) ‡				
NIH Stroke Scale (/42), calculated [40]				
Clinical deterioration (%) †				
Neurological deterioration (%)				
Headache (%)				
Hypotension (%)				
Hypertension (%)				
Venous thromboembolism (%)				

Hospital events
Hospital stay (days)
Discharged home (%)

Day 90
Death (%)
Barthel Index (BI, /100) ‡
Barthel Index <60 (%)
Zung Depression Scale (ZDS, /100) ‡
EQ-5D Health Utility Status (HUS, /1) ‡
EQ-Visual Analogue Scale (EQ-VAS, /100) ‡

† Neurological deterioration: decrease in SSS >4 points and/or decrease in consciousness on SSS >2 points; ‡: Death assigned, SSS -1, BI -5, ZDS 102.5, HUS 0, VAS -1

4 Table 4. Serious adverse events

4.1 Both GTN/no GTN and Continue/Stop publications

Table 4. Serious adverse events up to day 90. Data are number (%) of affected patients. Comparison by binary logistic regression.

Number (%)	Median time to event (days)	All			Fatal		
Cause		GTN	No GTN	2p	GTN	No GTN	2p
Cause		Continue	Stop	2p	Continue	Stop	2p
Neurological							
Complication of initial stroke							
Extension of initial stroke							
Haemorrhagic transformation of infarct							
Intracerebral bleed							
Recurrent stroke							
Cardiac							
Cardiac failure							
Hypertension							
Hypotension							
Myocardial infarction							
Sudden cardiac death		-	-	-			
Gastrointestinal							
Bleed							
Infarction							
Respiratory							
Pneumonia							
Pulmonary embolism							
Other							
Death, unattended		-	-	-			
Extracranial bleeding							
Malignancy/cancer							
Septicaemia							
Urinary tract infection							
Renal impairment							
Electrolyte imbalance							

4.2 Continue vs stop pre-stroke antihypertensive medications

Table 4a. Specific serious adverse events up to day 90 by antihypertensive drug class (where >15% of patients were taking the class) and by number of drug classes (where >15% of patients were taking that number of drugs). Data are number (%) of affected patients; the percentage of affected patients across drug classes may exceed 100%. Comparison by binary logistic regression.

**Number (%)**	**Median time to event (days)**	**All**			**Fatal**		
**Cause**		**Continue**	**Stop**	**2p**	**Continue**	**Stop**	**2p**

Drug class
ACE-I or ARB
ß-receptor antagonist
Calcium channel blocker
Diuretic
Other BP classes †
Number of drugs (%)
1
2
3
4
>4

† Other BP classes: alpha-receptor antagonist, centrally acting drugs, renin inhibitor
Index stroke-related:
Any of: complication of initial stroke, extension of initial stroke, recurrent stroke, haemorrhagic transformation of stroke; intracerebral bleed, recurrent stroke.
If the analysis shows a treatment-related stroke, the comparison will then be performed for each of these components.

5 Table 5. Protocol violations

5.1 Both GTN/no GTN and Continue/Stop publications

Table 5. Protocol violations by treatment group. Data are number (%).

	GTN	No GTN
	Continue	Stop
Inclusion criteria		
Failure to obtain consent/assent		
Age <18 years		
Time >48 hours		
GCS <8 (/15)		
No limb weakness		
No SBP <220 or >140 mmHg		
Known non-stroke intracranial pathology		
No brain imaging or autopsy		
Treatment		
Received antihypertensive drug(s) prior to randomisation		

In addition, for GTN vs no GTN

	Continue	Stop
Treatment		
Failure to stop pre-stroke nitrate treatment		
Failure to receive GTN for ≥4 days if randomised to GTN		
Received any GTN during first 4 days if randomised to no GTN		

In addition, for continue versus stop pre-stroke antihypertensive drugs

	Continue	Stop
Inclusion criteria		
Enrolled but not on a pre-stroke antihypertensive agent		
Treatment		
Received usual antihypertensive drug(s) before randomisation		
Received usual or new antihypertensive drug(s) when randomised to stop		
Failure to receive pre-stroke antihypertensive drug(s) when randomised to continue		

6 Table A. Blood pressure

6.1 Both GTN/no GTN and Continue/Stop publications

Table A. Blood pressure, systolic/diastolic (mmHg) course over 7 days of treatment. Data are mean (standard deviation) [number]. Comparison by t test with adjustment for multiple comparisons using Bonferroni correction.

Day	GTN	No GTN	Difference (SBP/DBP)	2p
Day	Continue	Stop	Difference (SBP/DBP)	2p
0, baseline			-	-
1, 2 hours post patch				
2				
3				
4				
5				
6				
7				

Note: PLAN IS TO SHOW THIS AS A FIGURE BUT NUMBERS NEEDED IN FIRST INSTANCE

7 Table B. mRS by treatment assignment

7.1 Both GTN/no GTN and Continue/Stop publications

mRS	0	1	2	3	4	5	Death
GTN	N1 (%)	.	.	.	.	.	.
No GTN	.	.	.	.	.	.	.

8 Table C. Outcome by sub-groups

8.1 Both GTN/no GTN and Continue/Stop publications

Effect of GTN vs no GTN on mRS at day 90 in subgroups defined at baseline. Data are odds ratio (95% confidence intervals) and interaction test. Comparison by ordinal logistic regression.

Variable	GTN	No GTN	OR (95% CI)	Interaction p
**Variable**	**Continue**	**Stop**	**OR (95% CI)**	**Interaction p**
Age, ≤70				
Age, >70				-
Sex, female				
Sex, male				-
History of hypertension, no				
History of hypertension, yes				-
Stroke, ischaemic				
Stroke, ICH				-
Stroke syndrome, LACS				
Stroke syndrome, PACS				-
Stroke syndrome, POCS				-
Stroke syndrome, TACS				-
Stroke severity, SSS >40				
Stroke severity, SSS 30-40				-
Stroke severity, SSS <30				-
Systolic BP, ≤160				
Systolic BP, 161-180				-
Systolic BP, 181-200				-
Systolic BP, >200				-
AF, absent				
AF, present				-
Feeding, oral				
Feeding, not oral				-
Ipsilateral carotid stenosis, 0-49%				
Ipsilateral carotid stenosis, 50-69%				-
Ipsilateral carotid stenosis, 70-99%				-
Ipsilateral carotid stenosis, 100%				-
Time to randomisation, ≤6 hr				
Time to randomisation, 6.1-12				-
Time to randomisation, 12.1-24				-
Time to randomisation, 24.1-36				-
Time to randomisation, >36				-
Treatment, alteplase				
Treatment, no alteplase				-
All patients				

GTN vs no GTN paper

Variable	GTN	No GTN	OR (95% CI)	Interaction p

History of recent nitrate, no				
History of recent nitrate, yes				-
Pre-stroke BP medication, not relevant				
Pre-stroke BP medication, continue				
Pre-stroke BP medication, stop				

Continue vs stop pre-stroke BP medications paper

**Variable**	**Continue**	**Stop**	**OR (95% CI)**	**Interaction p**
BP class, ACE-I/ARA/RI				
BP class, ß-RA				-
BP class, CCB				
BP class, diuretic				
BP class, centrally acting				
BP class, other				
No. of BP drugs, 1				
No. of BP drugs, 2				
No. of BP drugs, 3				
No. of BP drugs, 4				
No. of BP drugs, >4				
Randomised to GTN				
Randomised to no GTN				

NOTE: These tables will be presented as a Forest plot.

9 Figure 1. Trial flow diagram

9.1 Both GTN/no GTN and Continue/Stop publications

Figure 1. CONSORT flow diagram of patient randomisation, outcome, and losses to follow-up. Screening for eligibility was not collected routinely. Data are number (%).

Randomised		
	↙	↘
Treatment allocation	GTN	No GTN
Treatment allocation	Continue	Stop
Number allocated (*safety population*)		
Baseline data completed		
Adherence to allocation	↓	↓
Any GTN or no GTN (*efficacy population*)		
First dose of GTN or no GTN		
≥ 4days of GTN or no GTN (*s*)		
All 7 days of GTN or no GTN		
Day 7 follow-up (end of treatment)	↓	↓
Death by day 7		
Day 7 assessment completed		
<4 days		
7-10 days (per protocol)		
>10 days		
Day 7 assessment missing		
Patient refused		
Logistical problem		
Other reason		
Hospital discharge or death	↓	↓
Death in hospital		
Hospital event form completed		
Before day 90 follow-up		
On day 90 follow-up		
Hospital event form missing		
Patient refused		
Logistical problem		
Other reason		
Day 90 follow-up (end of follow-up)	↓	↓
Death by day 90		
No vital status available		
Day 90 assessment completed		
<83 days		
83-97 days (per protocol)		
>97 days		
Day 90 mRS assessment missing		
Patient lost to follow-up		
Patient refused		
Logistical problem		
Other		

10 Figure 2. Blood pressure profile

10.1 Both GTN/no GTN and Continue/Stop publications

Figure 2. Blood pressure during 7 days of treatment. ΔSBP and ΔDBP signify mean difference in systolic and diastolic blood pressure between the two groups; p values were calculated with the independent sample *t* test, and are for difference in systolic blood pressure between groups.

See table A for source data.

11 Figure 3. Modified Rankin Scale distribution

11.1 Both GTN/no GTN and Continue/Stop publications

Figure 3. Modified Rankin Scale (mRS) at 90 days follow-up. Distribution of mRS scores by treatment group. Comparison by ordinal logistic regression adjusted for baseline factors.

See table B for source data.

12 Figure 4. Primary outcome in pre-specified subgroups

12.1 Both GTN/no GTN and Continue/Stop publications

Figure 4. Subgroup analysis of effects on functional outcome at 90 days. Pre-defined subgroups are listed in section 4.4.3.

See table C for source data.

13 Figure 5. Survival curve

13.1 Both GTN/no GTN and Continue/Stop publications

Figure 5. Cumulative hazard of death during the 90 days of follow-up after randomisation. Cox proportional regression with adjustment for baseline covariates, hazard ratio= (95% CI -), p= .

14 Figure 6. Meta-analysis of relevant trials

14.1 GTN/no

Figure 6. Meta-analysis of trials in acute stroke involving drugs which lower blood pressure and which included more than 100 patients: effect on death or dependency

Study or Subgroup	Experimental Events	Experimental Total	Control Events	Control Total	Weight	Odds Ratio M-H, Random, 95% CI	Year
**1.1.1 Earlier trials**							
BEST 1988	93	201	43	100	5.1%	1.14 [0.70, 1.85]	1988
ASCLEPIOS 1990	47	116	44	114	4.4%	1.08 [0.64, 1.84]	1990
INWEST 1994	138	195	56	100	4.8%	1.90 [1.15, 3.14]	1994
Norris 1994	39	90	42	79	3.5%	0.67 [0.37, 1.24]	1994
Kaste 1994	44	175	31	172	4.6%	1.53 [0.91, 2.56]	1994
VENUS 1995	63	223	57	225	6.4%	1.16 [0.76, 1.76]	1995
Squire 1996	32	69	32	63	2.8%	0.84 [0.42, 1.66]	1996
ACCESS 2003	45	173	35	166	4.8%	1.32 [0.79, 2.18]	2003
IMAGES 2004	826	1188	858	1198	17.3%	0.90 [0.76, 1.08]	2004
INTERACT-1 2008	95	203	95	201	7.1%	0.98 [0.66, 1.45]	2008
CHHIPS 2009	69	113	35	59	3.2%	1.08 [0.57, 2.04]	2009
SCAST 2011	348	1000	331	1004	16.7%	1.09 [0.90, 1.31]	2011
INTERACT-2 2013	719	1382	785	1412	19.3%	0.87 [0.75, 1.01]	2013
FAST-Mag 2014	0	0	0	0		Not estimable	2014
**Subtotal (95% CI)**		**5128**		**4893**	**100.0%**	**1.03 [0.92, 1.17]**	
Total events	2558		2444				
Heterogeneity: Tau² = 0.01; Chi² = 18.34, df = 12 (P = 0.11); I² = 35%							
Test for overall effect: Z = 0.55 (P = 0.58)							
**1.1.2 ENOS**							
**Subtotal (95% CI)**		**0**		**0**		**Not estimable**	
Total events	0		0				
Heterogeneity: Not applicable							
Test for overall effect: Not applicable							
**Total (95% CI)**		**5128**		**4893**	**100.0%**	**1.03 [0.92, 1.17]**	
Total events	2558		2444				
Heterogeneity: Tau² = 0.01; Chi² = 18.34, df = 12 (P = 0.11); I² = 35%							
Test for overall effect: Z = 0.55 (P = 0.58)							
[illegible]							

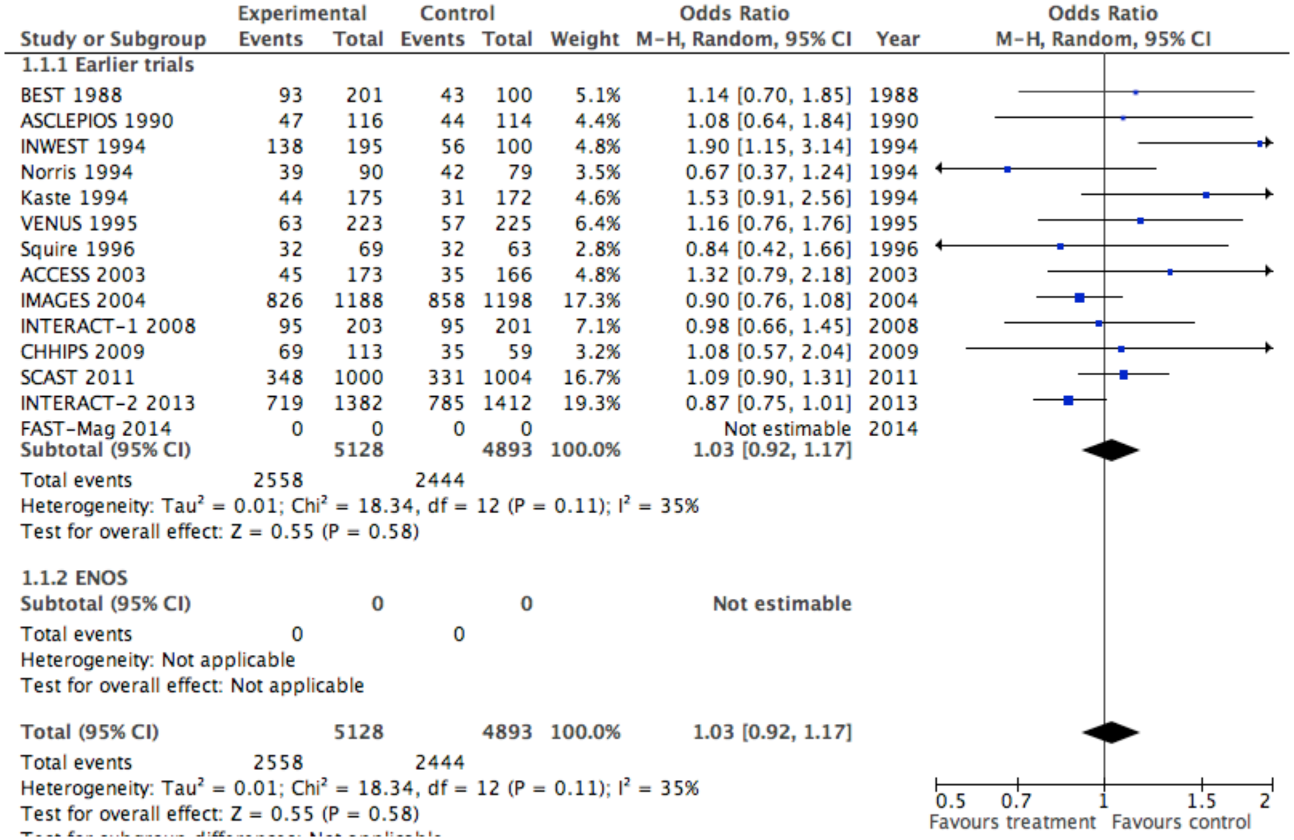

14.2 Continue/stop

Figure 6. Meta-analysis of trials in acute stroke comparing continuing versus stopping pre-stroke antihypertensive drugs and which included more than 100 patients: effect on death or dependency

Study or Subgroup	Continue Events	Continue Total	Stop Events	Stop Total	Weight	Odds Ratio M-H, Random, 95% CI
**2.1.1 Earlier trials**						
COSSACS 2010	142	379	141	384	100.0%	1.03 [0.77, 1.39]
**Subtotal (95% CI)**		379		384	100.0%	1.03 [0.77, 1.39]
Total events	142		141			
Heterogeneity: Not applicable						
Test for overall effect: Z = 0.21 (P = 0.83)						
**2.1.2 ENOS**						
ENOS 2014	0	0	0	0		Not estimable
**Subtotal (95% CI)**		0		0		Not estimable
Total events	0		0			
Heterogeneity: Not applicable						
Test for overall effect: Not applicable						
**Total (95% CI)**		379		384	100.0%	1.03 [0.77, 1.39]
Total events	142		141			
Heterogeneity: Not applicable						
Test for overall effect: Z = 0.21 (P = 0.83)						
Test for subgroup differences: Not applicable						

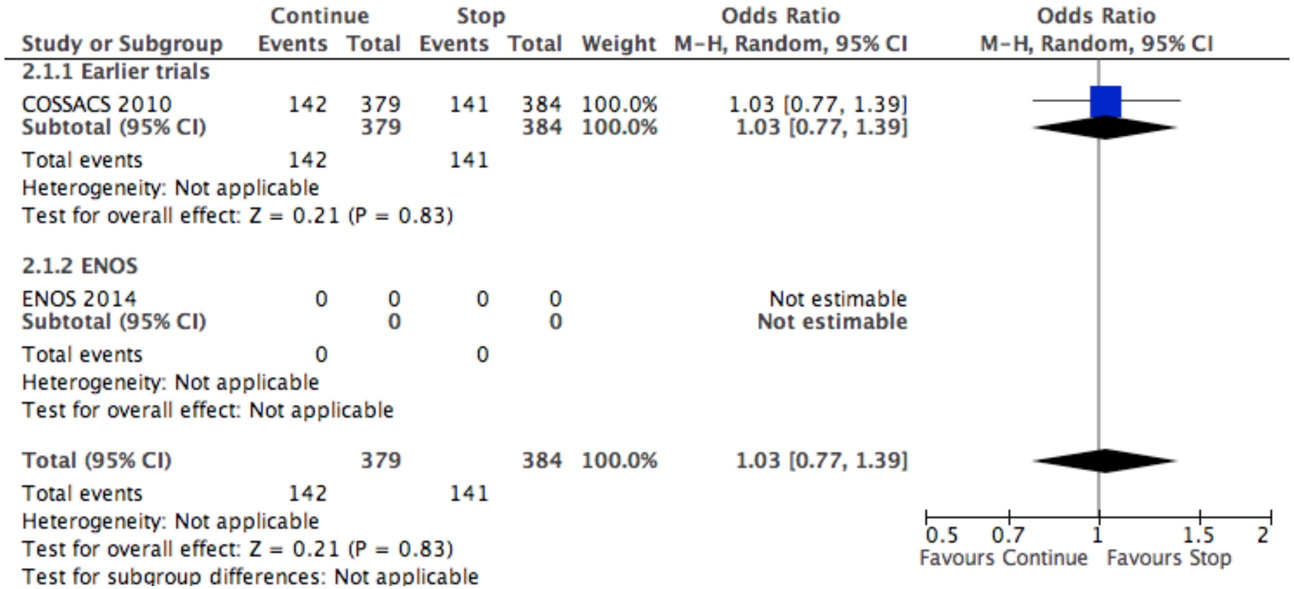

APPENDIX D. SECONDARY PUBLICATIONS

1 PUBLISHED

a. Protocol [15]
b. The NeuroGrid stroke exemplar clinical trial protocol [41]
c. Efficacy of Nitric Oxide in Stroke – a randomized trial. Characteristics of patients recruited in Poland[16]
d. The Efficacy of Nitric Oxide in Stroke (ENOS) trial – where do we stand in Romania? [20]
e. Safety and efficacy of lowering BP in patients with severe carotid stenosis [42]
f. Effect of the neutral CLOTS-1 trial on the use of graduated compression stockings in the Efficacy of Nitric Oxide Stroke (ENOS) trial [18]
g. Testing for differential item functioning within the EQ-5D [19]
h. Comparison of small volume infarcts of lacunar and non-lacunar aetiologies [43]

2 SUBMITTED

a. Relationship between cognitive impairment after stroke, baseline factors and functional outcome: Data from ENOS trial
b. Very low quality of life after acute stroke: data from the Efficacy of Nitric Oxide in Stroke (ENOS) trial. Stroke

3 IN PREPARATION

a. Methods for quantifying and qualifying intracerebral haemorrhage
b. Baseline characteristics. (To be submitted once last patient recruited. Appendix E)

4 PLANNED PUBLICATIONS

4.1 Treatment-related

a. The nitric oxide donor glyceryl trinitrate for treatment of acute stroke: the Efficacy of Nitric Oxide in Stroke (ENOS) randomised controlled trial (ISRCTN99414122). (Primary publication. Appendix C)
b. Continuing versus temporary stopping of pre-stroke antihypertensive therapy in patients with in acute stroke: the Efficacy of Nitric Oxide in Stroke (ENOS) randomised controlled trial (ISRCTN99414122). (Primary publication. Appendix C)
c. Glyceryl trinitrate and/or continuing pre-stroke antihypertensive medications in acute ischaemic stroke. (Appendix F)
d. Glyceryl trinitrate and/or continuing pre-stroke antihypertensive medications in acute spontaneous intracerebral haemorrhage. (Appendix G)
e. Glyceryl trinitrate for reducing cognition, quality of life and mood after stroke
f. Continuing versus stopping pre-stroke antihypertensive medication for reducing cognition, quality of life and mood after stroke
g. GTN-no GTN/Continue-stop and scan outcome at day 7 (Appendix E)
h. GTN, headache and outcome (Appendix F)
i. Effect of GTN on dysphagia (Appendix H)
j. Relationship between treatment-related change in blood pressure and outcome (as in SCAST [44])
k. Effect of GTN on haemodynamic parameters: systolic and diastolic BP, heart rate, pulse pressure, peak BP, BP variability, rate-pressure product and their

relationship to outcome (as in [45])

l. Health economics of GTN vs no GTN and/or Continue vs stop (if positive primary outcome)

4.2 'Epidemiological' (not treatment-related)

a. Comparison of functional outcomes between countries (as in [46-48])
b. Outcomes in UK
c. Identification of baseline phenotypic factors that predict cognitive outcomes

4.3 Systematic reviews/meta-analyses

a. Temporal effects of GTN on outcome, a meta-analysis of individual patient data from randomised controlled trials of GTN (Appendix M)
b. Comparison of continuing or temporary stopping pre-stroke antihypertensive medication: a prospective meta-analysis of individual patient data from randomised controlled trials (Appendix N). (A draft paper, based on merging data from ENOS, COSSACS,[49] and any other relevant trial(s), was written prior to the unblinding of COSSACS.)
c. Nitric oxide donors (nitrates), L-arginine, or nitric oxide synthase inhibitors in acute ischaemic stroke.[50] Update of this Cochrane Database of Systematic Review
d. Vasoactive drugs for acute stroke.[51] Update of this Cochrane Database of Systematic Review
e. Interventions for deliberately altering blood pressure in acute stroke.[52] Update of this Cochrane Database of Systematic Review
f. Blood pressure in Acute Stroke Collaboration: Intracerebral haemorrhage
g. Blood pressure in Acute Stroke Collaboration: Ischaemic stroke
h. Blood pressure in Acute Stroke Collaboration: All stroke

APPENDIX E. PLANNED PUBLICATION: BASELINE DATA

Authorship
To be decided.

Title
Characterisation of patients enrolled into the 'Efficacy of Nitric Oxide' in Stroke (ENOS) trial.

Authors
Members of Trial Steering Committee, International Advisory Committee and Trial Management Committee.

Aim
To characterise the patients enrolled into ENOS. For submission after enrolment of the last patient in quarter 4 2013.

Patients
All randomised patients.

Table 1
Recruitment: number of sites and patients by country

Country	No. of sites	No. of patients	%
Australia			
Canada			
China			
Denmark			
Egypt			
Eire			
Georgia			
Greece			
Hong Kong			
India			
Italy			
Malaysia			
Norway			
New Zealand			
Philippines			
Poland [16]			
Romania [20]			
Singapore [43]			
Sri Lanka			
Spain			
Sweden			
Turkey			
UK			
TOTAL			100

Table 2.
Clinical characteristics at baseline. Data are number (%), median [interquartile range] or mean (standard deviation).

	All patients	Continue-stop	Not in continue-stop
No. of patients			
Age (years)			
<18 (%) ¶			
18-40 (%)			
41-50 (%)			
51-60 (%)			
61-70 (%)			
71-80 (%)			
81-90 (%)			
91-100 (%)			
>100 (%)			
Mean (SD) †			
Sex			
Male (%) †			
Female (%)			
Geographical region ‡			
Africa			
America, north			
Asia, east			
Asia, south			
Asia, south-east			
Australasia			
British Isles			
Europe, mainland			
Medical history (%)			
Hypertension			
Treated hypertension ‡			
Hyperlipidaemia			
Diabetes mellitus			
Atrial fibrillation, current/previous			
Stroke			
Transient ischaemic attack			
Ischaemic heart disease			
Peripheral arterial disease			
Family history of young stroke, first degree relative			
Smoking (%)			
Current			
Past			
Never			
Alcohol, median [upw]			
>21 units per week			
<21			
None			
Pre-morbid modified Rankin Scale [/2]			
mRS 0			
mRS 1			
mRS 2			
mRS>2 ¶			
Medications (%)			
Blood pressure lowering			

Angiotensin converting enzyme inhibitor
Angiotensin receptor antagonist
Renin inhibitor
ß-receptor antagonist
Calcium channel blocker
Diuretic
α-receptor antagonist
Centrally acting
Other
Blood pressure tablets, number taken
Mean (SD)
Median [IQR]
Mode
0 (%)
1 (%)
2 (%)
3 (%)
4 (%)
>4 (%)
Lipid lowering
Nitrate
Side of lesion, right (%)
Scandinavian Stroke Scale (/58)
Mean (SD) †
Median [IQR]
51-58
41-50
31-40
21-30
11-20
0-10
NIHSS (/42), calculated [40]
Glasgow Coma Scale [/15]
15
14
13
12
11
10
9
<9 ¶
OCSP classification (%)
Total anterior †
Partial anterior
Lacunar
Posterior
TOAST classification (%) Ω∫
Cardioembolic
Large vessel
Small vessel/lacunar
Mixed
Unknown
Haemodynamics

Systolic blood pressure (mmHg)
<140 (mmHg)
140-160 (mmHg)
161-180 (mmHg)
181-200 (mmHg)
201-220 (mmHg)
>220 (mmHg)
Mean (SD) †
Diastolic blood pressure, mean (mmHg)
Heart rate, mean (bpm)
Temperature (°C)
Glucose (mmol/l)
Feeding (%)
Normal diet
Soft diet
Nasogastric fed
PEG-fed
Intravenous/subcutaneous fluids
Nil
Time stroke-randomisation (hours)
<1 (%) ¶
1-6 (%)
7-12 (%)
13-18 (%)
19-24 (%)
25-36 (%)
37-48 (%)
>48 (%) ¶
Median [IQR]
Mean (SD) †
Thrombolysis, given †
Qualifying event, by investigator (%) †
Ischaemic
Ischaemic stroke, no lesion on brain scan
Ischaemic stroke, lesion on brain scan
Haemorrhagic transformation of infarct
Not known
Intracerebral haemorrhage
Stroke type unknown
Non-stroke

† Minimisation variable; ‡ Stratification variable; Ω Ischaemic only; ∫ May exceed 100%; ¶ Protocol violation
bpm: beats per minute; NIHSS: National Institutes of Health Stroke Scale; TOAST: Trial of ORG 10172 in Acute Stroke Treatment

Table 3. Issues with the trial.

Issue	Explanation	Effect/Response
Sponsorship	Insurance and contracting issues prevented a number of countries from participating in ENOS.	Reduced world coverage and recruitment.
Recruitment, rate low	Recruitment limited by: Severe acute respiratory syndrome (SARS), Bird flu, competing trials, trial fatigue.	Delayed recruitment at various sites, especially in Singapore.
Recruitment, rate too high	Two sites over-recruited:<br>Site A: Outcome was poorly recorded, and had several losses to follow-up. A limited number of scans were available.<br>Site B: Review of baseline and follow-up data, consent forms, treatment charts and brain scans revealed no problem.	A: Site closed.<br>B: No action.
Recruitment, delayed	Tendency for patients to be recruited during day 2, in spite of repeated requests to investigators to focus on recruiting patients at the time of presentation to hospital.	Extended mean time to recruitment.
Randomisation to continue/stop	Patients not on pre-stroke antihypertensive drugs randomised in error (N=).	Sites training on inclusion criteria
Non-randomisation to continue/stop	Patients on pre-stroke antihypertensive drugs not randomised because drug(s) not recognised as an antihypertensive (N=).	Sites given full international list of anti-hypertensive agents.
Non-availability of placebo GTN patches	Placebo patches were made available by one manufacturer for the first pilot trial.[10] Pilot trials 2-4 used a single-blind design with GTN patch (or similar area of skin) covered by a gauze dressing.[11-13]	ENOS designed as single-blind study as for pilot trials 2-4.
Loss of availability of GTN patches	In China, GTN patches were sourced from a local manufacturer. A reduction in the national reimbursement for manufacturing these patches meant that the company ceased their production. Importation of patches from overseas probed too complicated to organise.	Cessation of recruitment in China.
Inappropriate GTN treatment	GTN given open-label to all patients at one site irrespective of randomisation.	Data from this site excluded from all analyses (exclusion in 2012, i.e. prior to data lock).
Theft of data	Theft of paper case report forms and brain scan films from one site relating to patients.	Missing follow-up and CT scan data.
Withdrawals	Withdrawal from follow-up (N=) or lost to follow-up (N=).	Missing primary outcome data.

Table 4. Univariate correlations between baseline characteristics for: age, sex, stroke severity (Scandinavian Stroke Scale, SSS), stroke pathological type (ischaemic, haemorrhage), systolic blood pressure (SBP), time to randomisation, and year of randomisation. Correlations by point biserial or Spearman's tests; data are regression coefficient and p value.

	Age	Sex, male	Severity (SSS)	Stroke type, bleed	Systolic BP	Time to randomisation	Year of randomisation
Age	X						
Sex		X					
Severity			X				
Type				X			
SBP					X		
Time						X	
Year							X

Figure 1. Recruitment curve throughout the trial.

Figure 2. Scatterplots and box and whisker plots showing univariate-relationships between baseline variables
Scatterplots and box-and-whisker plots of (i) age versus stroke severity (Scandinavian Stroke Scale, SSS); (ii) age vs systolic blood pressure (SBP); (iii) SSS vs SBP; (iv) Time stroke-randomisation vs SBP; (v) Time stroke-randomisation vs SSS.

APPENDIX A. ENOS protocol version 1.5 – latest/current version

APPENDIX B. ENOS protocol version 1.5 - signed front page.

APPENDIX F. PLANNED PUBLICATION: GLYCERYL TRINITRATE AND/OR CONTINUING PRE-STROKE ANTIHYPERTENSIVE MEDICATIONS IN ACUTE ISCHAEMIC STROKE

Authorship
Kailash Krishnan, …, Rob Dineen, Joanna Wardlaw, Philip Bath

Title
Glyceryl trinitrate and/or continuing pre-stroke antihypertensive medications in acute ischaemic stroke.

Authors
To include neuroimaging adjudicators.

Aim
To assess the effect of GTN vs no GTN, and continue vs stop temporarily pre-stroke antihypertensive medications, in patients with a final diagnosis of acute ischaemic stroke.

Patients
All randomised patients with acute ischaemic stroke, by intention-to-treat.

Layout
The publication will follow the format of the two primary publications (baseline characteristics, BP profile, clinical outcomes, SAEs, mRS distribution, survival) but will report the effect of both intervention comparisons (GTN vs no GTN, continue vs stop pre-stroke antihypertensive agents) in patients with acute ischaemic stroke as the final diagnosis. In addition, adjudicated information on baseline imaging data will be described (reporting the imaging variables in the table below), as will the effect of the interventions on day 7 imaging parameters (adjusted for baseline) where available (table).

Imaging data
The effect of the interventions on imaging parameters will be assessed where both baseline and day 7 data are present. The method of analysis is given for each imaging parameter.

Table 1. Baseline IS characteristics
Baseline demographic, clinical and neuroimaging characteristics of patients with acute ischaemic stroke. Data are number (%), median [interquartile range] or mean (standard deviation).

	All	GTN	No GTN	Continue	Stop
Patients (N)					
Country, UK (%)					
Age (years)					
Sex, male (%)					
Smoking, current (%)					
Pre-morbid mRS =0 (%)					
Previous stroke (%)					
Prior antihypertensive drug use (%)					
Prior history of high BP (%)					

Diabetes mellitus (%)					
Ischaemic heart disease (%)					
Atrial fibrillation (%)					
TACS (%)					
Scandinavian Stroke Scale (/58)					
Glasgow Coma Scale [/15]					
Systolic blood pressure (mmHg)					
Diastolic blood pressure (mmHg)					
Heart rate (bpm)					
Onset to neuroimaging (%)					
<12 hours					
12-24 hours					
>24 hours					
Location of infarct (%)					
Lobar					
Basal ganglia or thalamus					
Brainstem					
Cerebellum					
Undetermined					
Site					
Lesion size: MCA, lacunar, cerebellum or brainstem					
Mass effect, degree					
Haemorrhagic Transformation of Infarct					
Loss of grey-white definition					
Loss of basal ganglia outline					
Hypodensity					
Hypoattenuation					
Mass effect					
Mass effect score (greatest of Uncal> shift> ventricular> sulcal)					
Middle Cerebral Artery 1/3 (if MCA, >1/3, <1/3, no lesion)					
ASPECTS score					
Second lesion					
Hyperdense artery					
Leukoaraiosis					
Previous stroke lesion (%)					

bpm: beats per minute

Table 2. Effect of interventions on imaging outcomes at day 7
Effect of GTN vs no GTN, and continue vs stop temporarily pre-stroke antihypertensive agents, on imaging outcomes at day 7. Data are number (%), median (interquartile range) or mode. Comparisons by binary logistic regression or ordinal logistic regression, with adjustment for age; sex; history of hypertension; previous stroke; diabetes mellitus; current use of nitrate therapy (not for continue vs stop); stroke severity (SSS); total anterior circulation syndrome; systolic BP; treated with rt-PA prior to randomisation; feeding status; and time from onset to randomisation.

Imaging parameter	GTN	No	OR	p	Continue	Stop	OR	p

		GTN						
Patients (N)								
Recent stroke								
Site								
Lesion size: MCA, lacunar, cerebellum or brainstem								
Mass effect, degree								
Haemorrhagic transformation of infarct								
Loss of grey-white definition								
Loss of basal ganglia outline								
Hypodensity								
Hypoattenuation								
Mass effect								
Mass effect score (greatest of Uncal> shift> ventricular> sulcal)								
Middle cerebral artery 1/3 (if MCA, >1/3, <1/3, no lesion)								
ASPECTS score								
Second lesion								
Hyperdense artery								
PVL								
Atrophy								

Table 3. Effect of interventions on clinical outcomes
Effect of GTN vs no GTN, and continue vs stop temporarily pre-stroke antihypertensive agents, on clinical outcomes. Data are number (%), median (interquartile range) or mean (standard deviation). Comparisons by binary logistic regression, ordinal logistic regression or multiple linear regression, with adjustment for age; sex; history of hypertension; previous stroke; diabetes mellitus; current use of nitrate therapy (not for continue vs stop); stroke severity (SSS); total anterior circulation syndrome; systolic BP; alteplase; and time from onset to randomisation.

	All	GTN	No GTN	OR/ß	Continue	Stop	OR/ß
Day 7							
Patients (N)							
Death (%)							
Death or deterioration (%)							
SSS (/58)							
Recurrence (%)							
SBP (mmHg)							
Hospital events							
Patients (N)							
Death (%)							
Length of stay (days)							
Home (%)							
Day 90							
Patients (N)							
Death (%)							
Modified Rankin Scale (/6)							

Barthel Index (/100)							
tMMSE (/16)							
TICS (/39)							
Animal naming (/∞)							
ZDS (/100)							
HUS (EQ-5D) (/1)							
EQ-VAS (/100)							

Figure 1. Death by treatment group
Forest plot for hazard of death (calculated by Cox regression) with 95% confidence intervals at day 90 by pre-specified baseline covariates (as listed in section 4.4.3) for (a) GTN vs no GTN; and (b) continue vs stop pre-stroke antihypertensive medications.

Figure 2. Functional outcome by treatment group
Forest plot for odds ratio of mRS (calculated by ordinal logistic regression) with 95% confidence intervals at day 90 by pre-specified baseline covariates (as listed in section 4.4.3) for (a) GTN/no GTN; and (b) continue/stop pre-stroke antihypertensive medications.

Figure 3. Survival by treatment group
Kaplan-Meier plot for survival for (a) GTN/no GTN; and (b) continue/stop pre-stroke antihypertensive medications.

Figure 4. Functional outcome by treatment group in imaging subgroups
Forest plot for odds ratio of mRS (calculated by ordinal logistic regression) with 95% confidence intervals at day 90 for GTN/no GTN by pre-specified baseline imaging covariates: large lesion, hyperdense artery, atrophy, old lesions, WML

APPENDIX G. PLANNED PUBLICATION: GLYCERYL TRINITRATE AND/OR CONTINUING PRE-STROKE ANTIHYPERTENSIVE MEDICATIONS IN ACUTE SPONTANEOUS INTRACEREBRAL HAEMORRHAGE

Authorship
Kailash Krishnan, …, Rob Dineen, Niki Sprigg, Joanna Wardlaw, Philip Bath

Title
Glyceryl trinitrate and/or continuing pre-stroke antihypertensive medications in acute spontaneous intracerebral haemorrhage.

Authors
To include neuroimaging adjudicators and K Krishnan.

Aim
To assess the effect of GTN vs no GTN, and continue vs stop temporarily pre-stroke antihypertensive medications, in patients with a final diagnosis of acute spontaneous intracerebral haemorrhage.

Patients
All randomised patients with acute spontaneous intracerebral haemorrhage, by intention-to-treat.

Layout
The publication will follow the format of the two primary publications (baseline characteristics, BP profile, clinical outcomes, SAEs, mRS distribution, survival) but will report the effect of both intervention comparisons (GTN vs no GTN, continue vs stop pre-stroke antihypertensive agents) in patients with acute spontaneous intracerebral haemorrhage as the final diagnosis. In addition, adjudicated information on baseline imaging data will be described (reporting the imaging variables in the table below), as will the effect of the interventions on day 7 imaging parameters (adjusted for baseline) where available (table).

Imaging data
The effect of the interventions on imaging parameters will be assessed where both baseline and day 7 data are present. The method of analysis is given for each imaging parameter.

Table 1. Baseline ICH characteristics
Baseline demographic, clinical and neuroimaging characteristics of patients with acute spontaneous intracerebral haemorrhage. Data are number (%), median [interquartile range] or mean (standard deviation).

	All	GTN	No GTN	Continue	Stop
Patients (N)					
Country, UK (%)					
Age (years)					
Sex, male (%)					
Smoking, current (%)					
Pre-morbid mRS =0 (%)					
Previous stroke (%)					
Prior antihypertensive drug use (%)					
Prior history of high BP (%)					

Diabetes mellitus (%)					
Ischaemic heart disease (%)					
Atrial fibrillation (%)					
TACS (%)					
Scandinavian Stroke Scale (/58)					
Glasgow Coma Scale [/15]					
Systolic blood pressure (mmHg)					
Diastolic blood pressure (mmHg)					
Heart rate (bpm)					
Onset to neuroimaging (%)					
<12 hours					
12-24 hours					
>24 hours					
Location of haematoma (%)					
Lobar					
Basal ganglia or thalamus					
Brainstem					
Cerebellum					
Undetermined					
Longest diameter (cm)					
<3					
3-5					
5-8					
>8					
Leukoaraiosis					
Mass effect					
Remote ICH (%)					
Previous stroke lesion (%)					
Sub-arachnoid haemorrhage					
Sub-dural haemorrhage					
Extra-dural haemorrhage					
Measured CT scan findings					
Volume, ABC/2 (cm3)					
Diameter, max (cm)					
Diameter, max adjudicated (mode, cm)					
With IVH					
Graeb score (/12) [36]					
Modified Graeb score (/32) [37]					
Volume (ml)					
Without IVH					
Shape (/5) [53]					
Shape index					
Density (/5) [53]					
Mean density					
SD					
Density index					

bpm: beats per minute

Table 2. Effect of interventions on imaging outcomes at day 7
Effect of GTN vs no GTN, and continue vs stop temporarily pre-stroke antihypertensive agents, on imaging outcomes at day 7. Data are number (%), median (interquartile range) or mode. Comparisons by binary logistic regression, ordinal logistic regression or multiple linear regression, with adjustment for age; sex; history of hypertension; previous stroke; diabetes mellitus; current use of nitrate therapy (not for continue vs stop); stroke severity (SSS); total anterior circulation syndrome; systolic BP; baseline variable, and time from onset to randomisation.

Imaging parameter	GTN	No GTN	Difference (95% CI)	p	Continue	Stop	Difference (95% CI)	p
Patients (N)								
Volume (ABC/2), absolute (ml)								
Volume (ABC/2), relative (%)								
Diameter, max (cm)								
Diameter, max – adjudicated (cm)								
Mean radial expansion > 1mm								
No IVH, number (N)								
Shape [/5]								
Shape index								
Density [/5]								
Mean density								
Density index								
IVH, number (N)								
Graeb [/12]								
Modified Graeb [/32]								
IVH volume (ml)								

Table 3. Effect of interventions on clinical outcomes
Effect of GTN vs no GTN, and continue vs stop temporarily pre-stroke antihypertensive agents, on clinical outcomes. Data are number (%), median (interquartile range) or mean (standard deviation). Comparisons by binary logistic regression, ordinal logistic regression or multiple linear regression, with adjustment for age; sex; history of hypertension; previous stroke; diabetes mellitus; current use of nitrate therapy (not for continue vs stop); stroke severity (SSS); total anterior circulation syndrome; systolic BP; and time from onset to randomisation.

	All	GTN	No GTN	OR/ß	Continue	Stop	OR/ß
Day 7							
Patients (N)							
Death (%)							
Death or deterioration (%)							
SSS (/58)							
Recurrence (%)							
SBP (mmHg)							

Hospital events							
Patients (N)							
Death (%)							
Length of stay (days)							
Home (%)							
Day 90							
Patients (N)							
Death (%)							
Modified Rankin Scale (/6)							
Barthel Index (/100)							
tMMSE (/16)							
TICS (/39)							
Animal naming (/∞)							
ZDS (/100)							
HUS (EQ-5D) (/1)							
EQ-VAS (/100)							

Figure 1. Death by treatment group
Forest plot for hazard of death (calculated by Cox regression) with 95% confidence intervals at day 90 by pre-specified baseline covariates (as listed in section 4.4.3) for (a) GTN vs no GTN; and (b) continue vs stop pre-stroke antihypertensive medications.

Figure 2. Functional outcome by treatment group
Forest plot for odds ratio of mRS (calculated by ordinal logistic regression) with 95% confidence intervals at day 90 by pre-specified baseline covariates (as listed in section 4.4.3) for (a) GTN/no GTN; and (b) continue/stop pre-stroke antihypertensive medications.

Figure 3. Survival by treatment group
Kaplan-Meier plot for survival for (a) GTN/no GTN; and (b) continue/stop pre-stroke antihypertensive medications.

APPENDIX H. PLANNED PUBLICATION: HAEMODYNAMIC EFFECTS OF GTN VERSUS NO GTN, AND CONTINUE VERSUS STOP PRE-STROKE ANTIHYPERTENSIVE MEDICATIONS

Authorship
…, Philip Bath

Title
Effect of glyceryl trinitrate and/or continuing pre-stroke antihypertensive medications on blood pressure and other haemodynamic parameters, and relationship between changes and functional outcome.

Aim
To assess the effect of GTN vs no GTN, and continue vs stop temporarily pre-stroke antihypertensive medications, on blood pressure, heart rate and their derivatives in patients with acute stroke.

Patients
All randomised patients with acute stroke, by intention-to-treat. Haemodynamic measures will be calculated as in appendix B, section 2.2.

Table 1. Baseline haemodynamic parameters
Baseline haemodynamic parameters by treatment assignment. Data are number (%) or mean (standard deviation).

	All	GTN	No GTN	Continue	Stop
Patients (N)					
SBP, mean (mmHg)					
SBP, peak (mmHg)					
SBP, intra-visit variability (mmHg)					
SBP, CV (%)					
DBP, mean (mmHg)					
DBP, peak (mmHg)					
DBP, intra-visit variability (mmHg)					
DBP, CV (%)					
HE, mean (bpm)					
HR, peak (bpm)					
HR, intra-visit variability (bpm)					
HR, CV (%)					
Mean arterial pressure (MAP) (mmHg)					
Mean pressure (MP) (mmHg)					
Pulse pressure (PP) (mmHg)					
Pulse pressure index (PPI) (%)					
Rate-pressure product (RPP) (mmHg.bpm)					

Table 2. Effect of interventions on haemodynamic variables
Effect of GTN vs no GTN, and continue vs stop pre-stroke antihypertensive agents, on haemodynamic variables at day 1, and over the first 4 days of treatment. Comparison by multiple logistic regression adjusted for baseline.

	GTN	No GTN	Difference (95% CI)	p	Continue	Stop	Difference (95% CI)	p

Day 1								
Patients (N)								
SBP, mean (mmHg)								
SBP, peak (mmHg)								
SBP, intra-visit variability (mmHg)								
SBP, CV (%)								
DBP, mean (mmHg)								
DBP, peak (mmHg)								
DBP, intra-visit variability (mmHg)								
DBP, CV (%)								
HE, mean (bpm)								
HR, peak (bpm)								
HR, intra-visit variability (bpm)								
HR, CV (%)								
Mean arterial pressure (MAP) (mmHg)								
Mean pressure (MP) (mmHg)								
Pulse pressure (PP) (mmHg)								
Pulse pressure index (PPI) (%)								
Rate-pressure product (RPP) (mmHg.bpm)								
Days 0-4								
Patients (N)								
Mean SBP across days 1-4 (mmHg)								
Inter-visit Variability (SBP-inter SD) (mmHg)								
CV SBP across days 0-4 (%)								
SBP peak across days 0-4 (mmHg)								

Figure 1. Change in SBP by 6 treatment groups
Profile in mean SBP over 7 days of treatment by 6 treatment groups: a) GTN/continue, b) GTN/stop, c) no GTN/continue, d) no GTN/stop, e) GTN/no pre-stroke agent, f) no GTN/no pre-stroke agent. Data are mean (standard deviation). Comparison of area under curves with ANOVA with adjustment for multiple comparisons.

Figure 2. Outcome by change in SBP

Distribution in functional outcome (mRS) at day 90, by magnitude of change in mean SBP and variability between day 1 and 0, grouped as: increase/no change, and small decrease, moderate decrease, large decrease (with the latter 3 groups divided equally into tertiles). The 'small decrease' group will act as the reference.[54] Data are number (%). Comparison by ordinal logistic regression adjusted for age; sex; stroke severity (SSS); total anterior circulation syndrome; stroke pathological type (unknown, IS, ICH); treated with rt-PA prior to randomisation; and time from onset to randomisation.

mRS	0	1	2	3	4	5	Death
Change in SBP							
Increase/no							
Small decrease							
Moderate decrease							
Large decrease							
Change in variability							
Increase/no							
Small decrease							
Moderate decrease							
Large decrease							

APPENDIX I. PLANNED PUBLICATION: GTN, HEADACHE AND OUTCOME

Authorship
Kailash Krishnan, …, Philip Bath

Title
Relationship of GTN-related headache with outcome (as in PRoFESS – submitted)

Background
The PRoFESS mega-trial found that the headache that occurs with dipyridamole - a nitric oxide modifying drug – is associated with less recurrence after ischaemic stroke [publication submitted]. Since the headache that occurs with GTN is mediated by a similar mechanism, GTN-associated headaches may also be associated with subsequent outcome.

Hypotheses
- The presence of a headache by day 7 in patients taking GTN is associated with a better functional outcome and less recurrence than those with out a headache.
- The presence of a headache by day 7 in patients taking control is not associated with outcome.

Patients
All ENOS patients who received at least their first treatment (GTN/no GTN), who have information on headache (yes/no) at day 7, and who have functional outcome (mRS) recorded at day 90. SAEs for headache within the first 7 days will be validated against data for headache on the day 7 form.

Table 1. Baseline table
Baseline characteristics for patients with and without a headache by day 7, by treatment group. Data are number (%) or mean (standard deviation). Comparison by Chi-square or t test

	GTN		2p	No	GTN	2p
Headache	Yes	No		Yes	No	
N						
Age (years)						
Sex, male (%)						
Smoker, current (%)						
Alcohol, excess (%)						
Hypertension (%)						
Diabetes (%)						
Stroke (%)						
Ischaemic heart disease (%)						
Atrial fibrillation (%)						
Peripheral vascular disease (%)						
Severity (SSS, /58)						
TACS (%)						
SBP (mmHg)						
DBP (mmHg)						
Heart rate (bpm)						

Table 2. Outcome table
Relationship between outcome and presence of a headache by day 7, by treatment group. Data are number (%) or mean (standard deviation), and odds ratio or ß-

coefficient (95% confidence intervals). Comparison within treatment groups by binary logistic regression, ordinal logistic regression, multiple linear regression or Cox regression.

	GTN		OR/ß	No	GTN	OR/ß
Headache	Yes	No		Yes	No	
Day 7						
Patients (N)						
Death (%)						
Deterioration or death (%)						
Ischaemic stroke						
Recurrence (%)						
sICH (%)						
SSS (/58)						
Hospital events						
Patients (N)						
Length of stay (days)						
Death (%)						
Day 90						
Patients (N)						
Death (%)						
Modified Rankin Scale (/6)						
Barthel Index (/100)						
tMMSE (/16)						
TICS (/100)						
Animal naming (/∞)						
ZDS (/100)						
HUS (EQ-5D) (/1)						
EQ-VAS (/100)						

Figure 1. Survival
Survival curves for patients with and without headache by day 7, by treatment group. Comparison within treatment groups by Cox regression.

Figure 2. Functional outcome
Modified Rankin Scale at day 90 for patients with and without headache by day 7, by treatment group. Data are number (%). Comparison within treatment groups by ordinal logistic regression.

mRS	0	1	2	3	4	5	Death
GTN							
Headache							
No headache							
No GTN							
Headache							
No headache							

APPENDIX J. PLANNED PUBLICATION: EFFECT OF GTN ON FEEDING STATUS

Authorship
…, Shaheen Hamdy, David Smithard, David Cohen, … Niki Sprigg, Philip Bath

Title
Effect of transdermal glyceryl trinitrate on feeding status at day 7 after stroke, and relationship of feeding status with functional outcome: results from the 'Efficacy of Nitric Oxide in Stroke' (ENOS) trial.

Hypothesis
GTN relaxes smooth muscle and therefore may improve the oesophageal phase of swallowing. A small/pilot randomised trial of nifedipine, a calcium channel blocker that also relaxes oesophageal smooth muscle, reported potentially beneficial effects.[55]

Patients
Patients in ENOS with information on feeding status at both baseline and day 7, and a final diagnosis of strokes. Analysis by intention-to-treat.
Feeding status is defined on a 6-level ordered categorical scale (normal diet, soft diet, nasogastric tube fed, PEG-tube fed, iv/sc fluids, no feeding/fluids).
Since dysphagia was not directly assessed, dysphagia is defined clinically as patients who cannot take fluids/food orally, i.e.:
- Not dysphagic/oral feeding: normal diet, soft diet
- Dysphagic/no oral feeding: NGT-feeding, PEG/RIG-feeding, iv/sc fluids or no feeding/fluids
- Missing data - No information on feeding status

Table 1. Baseline data
Characteristics of patients with and without clinical dysphagia at baseline. Data are number (%) or mean (standard deviation). Comparison of patients who have versus do not have oral feeding by Chi-square test or t test.

	All	GTN	No GTN	No oral feeding	Oral feeding	Difference (95% CI)	2p
Number of participants							
Age (years)							
Sex, male (%)							
Stroke, previous (%)							
Hypertension (%)							
Ischaemic heart disease (%)							
Scandinavian Stroke Scale (/58)							
Systolic blood pressure (mmHg)							
Ischaemic stroke (%)							
Time to randomisation (hours)							
Stroke syndrome (%)							
TACS							
PACS							
POCS							
LACS							
Stroke type, ischaemic (%)							

Feeding status (%)							
Normal diet				-			-
Soft diet				-			-
NGT-fed					-		-
PEG-fed					-		-
Intravenous/subcutaneous fluids					-		-
No feeding fluids					-		-

Table 2a. Change in feeding status
Change in feeding status from baseline to day 7 for patients randomised to GTN. Data are number (%).

Baseline	Normal diet	Soft diet	NGT-fed	PEG-fed	Iv/sc fluids	No feeding/fluids	Missing data
Day 7							
Normal diet							
Soft diet							
NGT-fed							
PEG-fed							
Iv/sc fluids							
No feeding/fluids							
Death							
Missing data							

Table 2b. Change in feeding status
Change in feeding status from baseline to day 7 for patients randomised to no GTN. Data are number (%).

Baseline	Normal diet	Soft diet	NGT-fed	PEG-fed	Iv/sc fluids	No feeding/fluids	Missing data
Day 7							
Normal diet							
Soft diet							
NGT-fed							
PEG-fed							
Iv/sc fluids							
No feeding/fluids							
Death							
Missing data							

Table 3 Outcomes by GTN/no GTN
Effect of GTN vs no GTN on feeding status at day 7 in subgroups defined at baseline. Data are odds ratio (95% confidence intervals) and interaction test. Comparison by ordinal logistic regression. This table may be presented as a Forest plot.

Variable	GTN	No GTN	OR (95% CI)	Interaction p
Age, ≤70				
Age, >70				-

Sex, female				
Sex, male				-
Previous stroke, no				
Previous stroke, yes				-
Stroke syndrome, LACS				
Stroke syndrome, PACS				-
Stroke syndrome, POCS				-
Stroke syndrome, TACS				-
Stroke severity, SSS >40				
Stroke severity, SSS 30-40				-
Stroke severity, SSS <30				-
Stroke, ischaemic				
Stroke, ICH				-
Feeding, oral				
Feeding, not oral				-
Time to randomisation, ≤6				
Time to randomisation, 6.1-12				-
Time to randomisation, 12.1-24				-
Time to randomisation, 24.1-36				-
Time to randomisation, 36.1-48				-
Treatment, alteplase				
Treatment, no alteplase				-
All patients				

Table 4. Outcomes by dysphagia/no dysphagia
Comparison of outcomes at days 7 and 90 by swallowing status at baseline. Data are number (%) or mean (standard deviation), and odds ratio/ß-coefficient (95% confidence intervals). Comparison by binary logistic regression, ordinal logistic regression, or multiple linear regression, with adjustment for age, sex, previous stroke, severity, stroke pathological type, time, treatment assignment (GTN/no GTN).

	N	All patients	No oral feeding	Oral feeding	OR/ß
Day 7					
Patients (N)					
Death (%)					
Death or deterioration (%)					
SSS (/58)					
Recurrence (%)					
SAEs, all (%)					
Pneumonia					
Hospital events					
Patients (N)					
Death (%)					
Length of stay (days)					
Home (%)					
Day 90					
Patients (N)					
Death (%)					
Modified Rankin Scale (/6)					

Barthel Index (/100)					
tMMSE (/16)					
TICS (/39)					
Animal naming (/∞)					
ZDS (/100)					
HUS (EQ-5D) (/1)					
EQ-VAS (/100)					
SAEs, all (%)					
Pneumonia					

Figure 1. Feeding status at day 7
Shift in feeding status at day 7 by treatment with GTN vs no GTN. Data are number (%). Comparison by ordinal logistic regression adjusted for age, sex, stroke severity (SSS), TACS, stroke pathological type (unknown, IS, ICH), SBP, time between stroke and randomisation, and baseline feeding status.

Feeding status	Normal diet	Soft diet	NGT-fed	PEG-fed	Iv/sc fluids	No feeding/fluids
GTN						
No GTN						

Figure 2. Modified Rankin Scale at day 90
Shift in modified Rankin Scale at day 90 between patients who were dysphagic (no oral feeding) versus those who were not (oral feeding). Data are number (%). Comparison by ordinal logistic regression adjusted for age, sex, stroke severity (SSS), TACS, stroke pathological type (ICH, ischaemic), SBP, time for onset to randomisation, baseline feeding status and treatment assignment.

mRS	0	1	2	3	4	5	Death
Dysphagia							
No dysphagia							

Figure 3. Dysphagia in sub-groups
Forrest plot of feeding status in pre-specified sub-groups, as listed in table 3.

Figure 4. Survival
Survival curve for dysphagia vs no dysphagia for (a) all patients to 100 days, and (b) UK patients to 4000 days. Comparisons by Cox regression adjusted for age, sex, stroke severity (SSS), TACS, stroke pathological type (ICH, ischaemic), SBP, time between stroke and randomisation, baseline feeding status and treatment assignment.

APPENDIX K. PLANNED PUBLICATION: SPONTANEOUS INTRACEREBRAL HAEMORRHAGE BETWEEN DIFFERENT WORLD REGIONS

Authorship
Kailash Krishnan, …, Niki Sprigg, Joanna Wardlaw, Philip Bath

Title
Comparison of acute spontaneous intracerebral haemorrhage between different world regions: patients, haematoma and outcomes in the ENOS trial.

Authors
To include neuroimaging adjudicators and K Krishnan.

Aim
To compare patients and haematoma characteristics, and outcomes, between different regions of the world. Approaches used in previous publications comparing stroke outcomes between countries [46,48,56] will be followed.

Patients
All randomised patients with acute spontaneous intracerebral haemorrhage, by intention-to-treat:
a) Africa: Egypt
b) America, north: Canada
c) Asia, east: China, Hong Kong
d) Asia, south: India, Sri Lanka
e) Asia, south-east: Malaysia, Philippines, Singapore
f) Australasia: Australia, New Zealand
g) British Isles: Eire, UK
h) Europe: Denmark, Georgia, Greece, Italy, Norway, Poland, Romania, Spain, Sweden

Table 1. Baseline ICH characteristics
Baseline demographic, clinical and neuroimaging characteristics of patients with spontaneous intracerebral haemorrhage. Data are number (%), median [interquartile range] or mean (standard deviation).

	All	Africa	America, north	Asia east	Asia south	Asia south-east	Austral-asia	British Isles	Europe
Patients (N)									
Age (years)									
Sex, male (%)									
Smoking, current (%)									
Pre-morbid mRS =0 (%)									
Previous stroke (%)									
Prior antiHT drug use (%)									
Prior history of high BP									

(%)									
Diabetes mellitus (%)									
Ischaemic heart disease (%)									
Atrial fibrillation (%)									
TACS (%)									
Scandinavian Stroke Scale (/58)									
Glasgow Coma Scale [/15]									
SBP (mmHg)									
DBP (mmHg)									
HR (bpm)									
Onset to neuroimaging (%)									
<12 hours									
12-24 hours									
>24 hours									
Adjudicated parameters									
Location of haematoma (%)									
Lobar									
Basal ganglia or thalamus									
Brainstem									
Cerebellum									
Longest diameter (cm)									
<3									
3-5									
5-8									
>8									
Leukoaraiosis									
Mass effect									
Remote ICH (%)									
Previous stroke lesion (%)									
Sub-									

arachnoid haemorrhage									
Sub-dural haemorrhage									
Extra-dural haemorrhage									
Measured CT scan findings									
Volume, ABC/2 (cm3)									
Diameter, max (cm)									
Diameter, max adjudicated (mode, cm)									
With IVH									
Graeb score (/12) [36]									
Volume (ml)									
Without IVH									
Shape (/5) [53]									
Density (/5) [53]									

bpm: beats per minute

Table 2. Functional outcome
Comparison of functional outcome at day 90 by world region. Data are number (%), median (interquartile range) or mean (standard deviation. Comparisons Chis-square test or Kruskal-Wallis test. by binary logistic regression, ordinal logistic regression or multiple linear regression, with adjustment for age; sex; history of hypertension; previous stroke; diabetes mellitus; stroke severity (SSS); total anterior circulation syndrome; systolic BP; ICH volume; time from onset to randomisation; GTN/no GTN; and continue/stop.

	All	Africa	America, north	Asia east	Asia south	Asia south-east	Austral-asia	British Isles	Europe	p
Day 7										
Patients (N)										
Death (%)										
Death or deterioration (%)										
Recurrence (%)										
SSS (/58)										
SBP										

(mmHg)										
SAEs (%)										
Cerebral oedema										
Hospital events										
Patients (N)										
Death (%)										
Home (%)										
Length of stay (days)										
Day 90										
Death (%)										
mRS (/6)										
BI (/100)										
tMMSE (/16)										
TICS (/39)										
Animal naming (/∞)										
ZDS (/100)										
HUS (EQ-5D) (/1)										
EQ-VAS (/100)										

Figure 1. Death by country
Forest plot for hazard of death (calculated by Cox regression) with 95% confidence intervals at day 90 by geographical region relative to the British Isles. For analyses (a) unadjusted; and (b) adjusted for age; sex; history of hypertension; previous stroke; diabetes mellitus; stroke severity (SSS); total anterior circulation syndrome; systolic BP; ICH volume; time from onset to randomisation; GTN/no GTN; and continue/stop.

Figure 2. Functional outcome by country
Forest plot for odds ratio of mRS (calculated by ordinal logistic regression) with 95% confidence intervals, at day 90 by geographical region relative to the British Isles. For analyses (a) unadjusted; and (b) adjusted for age; sex; history of hypertension; previous stroke; diabetes mellitus; stroke severity (SSS); total anterior circulation syndrome; systolic BP; ICH volume; time from onset to randomisation; GTN/no GTN; and continue/stop.

Figure 3. Survival
Kaplan-Meier plot for survival by geographical region.

APPENDIX L. PLANNED PUBLICATION: COMPARISON OF PATIENTS WHO TOOK VERSUS DID NOT TAKE PRE-STROKE ANTIHYPERTENSIVE MEDICATIONS: BASELINE CHARACTERISTICS AND OUTCOMES

Authorship
…

Title
Comparison of patients who took versus did not take pre-stroke antihypertensive medications: baseline characteristics and outcomes.

Hypothesis
Patients taking pre-stroke antihypertensive medications will have more vascular risk factors and a worse outcome than those not on such medications.

Patients
Patients with confirmed stroke, by intention to treat.

Table 1. Baseline characteristics of included trials
Description of the trials and their participants. Trials are ordered by mean time to recruitment. Data are number (%) or mean (standard deviation).

Table based on baseline data publication.

Table 2. Outcomes
Comparison of outcomes in patients taking versus not taking pre-stroke antihypertensive medications.

	Analysis			OR/ß
Pre-stroke antihypertensive medications		Yes	No	
Day 7				
Patients (N)				
Death (%)				
Death or deterioration (%)				
Ischaemic stroke				
Recurrence (%)				
sICH (%)				
SSS (/58)				
Hospital events				
Patients (N)				
Death (%)				
Length of stay (days)				
Day 90				
Patients (N)				
Death (%)				
Modified Rankin Scale (/6)				
Barthel Index (/100)				
tMMSE (/16)				
TICS				
Animal naming (/∞)				
ZDS (/100)				
HUS (EQ-5D) (/1)				

EQ-VAS (/100)				

Figure 1. Modified Rankin Scale at day 90
Shift in modified Rankin Scale at day 90 between patients who took versus did not take pre-stroke antihypertensive medications. Data are number (%). Comparison by ordinal logistic regression adjusted for age, sex, geographical region, hypertension, hyperlipidaemia, diabetes mellitus, atrial fibrillation, previous stroke, previous TIA, ischaemic heart disease, smoking, pre-morbid mRS, stroke severity (SSS), GCS, TACS, stroke pathological type (ICH, ischaemic), SBP, time for onset to randomisation, baseline feeding status, and GTN vs no GTN.

mRS	0	1	2	3	4	5	Death
Yes							
No							

Figure 2. Survival by day 90
Survival curve between patients who took versus did not take pre-stroke antihypertensive medications. Comparisons by Cox regression adjusted for age, sex, geographical region, hypertension, hyperlipidaemia, diabetes mellitus, atrial fibrillation, previous stroke, previous TIA, ischaemic heart disease, smoking, pre-morbid mRS, stroke severity (SSS), GCS, TACS, stroke pathological type (ICH, ischaemic), SBP, time for onset to randomisation, baseline feeding status, and GTN vs no GTN.

APPENDIX M. PLANNED PUBLICATION: TEMPORAL EFFECTS OF GTN ON FUNCTIONAL OUTCOME

Authorship

…, Sandeep Ankolekar, Tim England, Kailash Krishnan, Parveen Rashid, Gill Sare, Niki Sprigg, Mark Willmot, Philip Bath

Title

Effect of transdermal glyceryl trinitrate on functional outcome in acute stroke by time to treatment: an individual patient data meta-analysis of all ultra-acute, acute, and subacute randomised trials of GTN.

Hypothesis

The effect of GTN on functional outcome may vary by time to treatment, as with alteplase.[57]

Patients

All patients in GTN-1/2/3, RIGHT, ENOS and any other identified randomised controlled trials of GTN,[10-13] by intention to treat. The publication will follow the approaches published for alteplase.[57,58]

Table 1. Baseline characteristics of included trials

Description of the trials and their participants. Trials are ordered by mean time to recruitment. Data are number (%) or mean (standard deviation).

Trial	All	GTN	No GTN	GTN-1[10]	GTN-2[11]	GTN-3[12]	RIGHT[13,14]	ENOS[15]
GTN dose								
Onset (hr)								
Number								
Age (years)								
Sex, male (%)								
Previous stroke (%)								
Hypertension (%)								
Diabetes mellitus (%)								
Ischaemic heart disease (%)								
Atrial fibrillation (%)								
Severity (SSS, /58)								
SBP (mmHg)								
DBP (mmHg)								
Heart rate (bpm)								
Ischaemic stroke (%)								
OTR (hr)								

Table 2. Outcome by time

Effect of GTN vs no GTN on outcomes by time epochs from stroke onset to randomisation (OTR). Data are odds ratio or beta coefficients (with 95% confidence intervals). Comparison between GTN vs no GTN by binary logistic regression, ordinal logistic regression, multiple regression or Cox regression, with adjustment for age, sex, severity (Scandinavian Stroke Scale), stroke pathological type (ischaemic, ICH), systolic blood pressure.

OTR (hr)	All	≤4	4.1-8	8.1-12	12.1-24	24.1-48	48.1-96	>96
End-of-treatment								
Patients (N)								
Death (%)								
Death/deterioration (%)								
Recurrence (%)								
SSS (/58)								
Discharge								
Patients (N)								
Death (%)								
Home (%)								
Length of stay (days)								
Day 90								
Death (%)								
mRS (/6)								
BI (/100)								
tMMSE (/16)								
TICS (/39)								
Animal naming (/∞)								
ZDS (/100)								
HUS (EQ-5D) (/1)								
EQ-VAS (/100)								

Table 3. Numbers-needed-to-treat by time
Numbers-needed-to-treat [59] by time epochs from stroke onset to randomisation (OTR). Data are NNT (with 95% confidence intervals) with adjustment for age, sex, severity (Scandinavian Stroke Scale), stroke pathological type (unknown, IS, ICH), systolic blood pressure.

OTR (hr)	All	≤4	4.1-8	8.1-12	12.1-24	24.1-48	48.1-96	>96
Day 90								
Death								
mRS								

Figure 1. Meta-analysis of trials
Forrest plot of meta-analysis of GTN trials.

Figure 2. Outcome by time-to-treatment
Relationship between outcome at day 90 and time from onset to randomisation, for (a) modified Rankin Scale (odds ratio for 7 level scale); (b) death (odds ratio); (c) Zung Depression Scale (ß-coefficient); and (d) Health Utility Status (derived from EQ-5D, ß-coefficient).

Figure 3. Functional outcome
Distribution of modified Rankin Scale for GTN vs no GTN by time from stroke to randomisation for time epochs (hours): ≤4, 4.1-8, 8.1-12, 12.1-24, 24.1-48, 48.1-96, >96.

APPENDIX N. PLANNED PUBLICATION: CONTINUING VERSUS STOPPING PRE-STROKE ANTIHYPERTENSIVE MEDICATIONS IN ACUTE STROKE: A META-ANALYSIS OF INDIVIDUAL PATIENT DATA FROM RANDOMISED CONTROLLED TRIALS

Authorship
Tom Robinson, …, Ken Lees, Philip Bath

Title
Comparison of continuing or temporary stopping of pre-stroke antihypertensive medication in acute stroke: a prospective meta-analysis of individual patient data from randomised controlled trials.

Hypothesis
Continuing pre-stroke antihypertensive therapy lowers BP and reduces poor functional outcome as opposed to temporary stopping of such treatment.

Patients
Patients recruited into trials comparing continuing versus stopping pre-stroke antihypertensive agents in patients with acute stroke, with analysis by intention to treat. The publication will follow the approaches published for alteplase.[57,58]

Table 1. Included trials
Characteristics of trials comparing continuing versus stopping pre-stroke antihypertensive medication in patients with acute stroke.

	Size	Recruitment time window (hrs)	Length of treatment (days)	Length of follow-up (days)	SBP range (mmHg)	Major exclusions
COSSACS [49]	763	< 48	14	180	>100	Need for antihypertensive agents
ENOS [15]	?	<48	7	90	140-220	Need for GTN or antihypertensive agents
Other trial(s)?						

GTN: glyceryl trinitrate

Table 2. Baseline characteristics
Baseline characteristics of trial participants. Data are number (%) or mean (standard deviation). Comparison by Chi-square test or ANOVA.

	COSSACS 49	ENOS 15	Other trial(s) ?	P-value
No. of patients				
Age (years)				
Sex, male (%)				
Race-ethnicity (%) [1]				
Caucasian				
Asia, east				
Asia, south				
Asia, south-east				
Other				
Medical history (%)				
Hypertension				
Diabetes mellitus				
Hyperlipidaemia				
Atrial fibrillation				
Previous stroke				
TIA				
Ischaemic heart disease				
Family history of stroke				
Smoker, current				
Rankin Scale, pre-morbid 0				
Antihypertensive agents pre-stroke				
Angiotensin converting enzyme inhibitor				
Angiotensin receptor antagonist				
Renin inhibitor				
ß-receptor antagonist				
Calcium channel blocker				
Diuretic				
α-receptor antagonist				
Centrally acting				
Other				
Number of antihypertensive agents				
1				
2				
3				
4				
>4				
Time since last BP drugs				
Medications, other pre-stroke				
Aspirin				
Dipyridamole				
Clopidogrel				
Statin				
Haemodynamic measures				
Systolic BP (mmHg)				
Diastolic BP (mmHg)				

Pulse pressure (mmHg)				
Systolic BP, peak (mmHg)				
Systolic BP, standard deviation (mmHg)				
Heart rate (bpm)				
Rate-pressure product (mmHg.bpm)				
Stroke severity, NIHSS [2]				
Stroke type/aetiology (%)				
IS [3]				
ICH				
Stroke type unknown				
Non stroke				
Stroke syndrome (%)				
Total anterior circulation				
Partial anterior circulation				
Posterior circulation				
Lacunar				
Stroke aetiology (if ischaemic) (%)				
Small vessel				
Large artery				
Cardioembolic				
Other				
Carotid stenosis, ipsilateral 70-99% [4]				
Time to randomisation (hr) (%)				
≤12				
13-24				
25-48				

1. ENOS: Race-ethnicity defined by country of recruitment
2. ENOS: NIHSS estimated from Scandinavian Stroke Scale [35]
3. Infarct: normal imaging or infarct with/without haemorrhagic transformation
4. COSSACS: Symptomatic carotid stenosis from SAE reporting carotid endarterectomy

Table 3. Outcomes
Functional outcome and vascular events. Percentage for continue versus stop; comparison by logistic regression, or multiple regression, adjusted for trial, age, sex, severity, systolic blood pressure.

Outcome	COSSACS		ENOS		Other trial?		All stroke OR (95% CI)	P-value	IS OR (95% CI)	P-value	ICH OR (95% CI)	P-value
	Continue	Stop	Continue	Stop	Continue	Stop						
Patients												
End of treatment [1]												
Death (%)												
Recurrence (%)												
Ischaemic												
Haemorrhagic												
Unknown												
Death or deterioration (%)												
Impairment, NIHSS [2]												
Systolic BP												
Diastolic BP												
End of trial [3]												
Death (%)												
mRS>2 (%)												
Stroke (%) [4]												
Myocardial infarction (%) [4]												
Vascular event (%) [5]												
Lost to follow-up (%)												

1. COSSACS: 14 days; ENOS: 7 days
2. ENOS: NIHSS estimated from Scandinavian Stroke Scale [40]
3. COSSACS: 180 days; ENOS: 90 days

4. ENOS (end of follow-up): Stoke and MI rates obtained from serious adverse events
5. Composite of vascular death, non-fatal stroke, and non-fatal myocardial infarction

Blood pressure profile

Figure 1. Blood pressure during 7 days of treatment. ΔSBP and ΔDBP signify mean difference in systolic and diastolic blood pressure between the two groups; p values were calculated with the independent sample *t* test, and are for difference in systolic blood pressure between groups.

Modified Rankin Scale distribution

Figure 2. Modified Rankin Scale (mRS) at 90 days follow-up. Distribution of mRS scores by treatment group. Comparison by ordinal logistic regression adjusted for baseline factors.

Primary outcome in pre-specified subgroups

Figure 3. Subgroup analysis of effects on functional outcome at 90 days. Pre-defined subgroups are listed in section 4.4.3.

Survival curves

Figure 4. Cumulative hazard of (a) death; (b) recurrence; and (c) cardiovascular events during follow-up. Cox proportional regression with adjustment for baseline covariates, hazard ratio= (95% CI -), p= .

REFERENCES

1. Leonardi-Bee J, Bath PMW, Phillips SJ, Sandercock PAG, for the IST Collaborative Group. Blood pressure and clinical outcomes in the International Stroke Trial. Stroke 2002;33:1315-20.
2. Willmot M, Leonardi-Bee J, Bath PM. High blood pressure in acute stroke and subsequent outcome: a systematic review. Hypertension 2004;43:18-24.
3. Sprigg N, Gray LJ, Bath PM, et al. Relationship between outcome and baseline blood pressure and other haemodynamic measures in acute ischaemic stroke: data from the TAIST trial. J Hypertens 2006;24:1413-7.
4. Geeganage C, Tracy M, England T, et al. Relationship Between Baseline Blood Pressure Parameters (Including Mean Pressure, Pulse Pressure, and Variability) and Early Outcome After Stroke: Data From the Tinzaparin in Acute Ischaemic Stroke Trial (TAIST). Stroke 2010:23.
5. Sandset EC, Bath PM, Boysen G, et al. The angiotensin-receptor blocker candesartan for treatment of acute stroke (SCAST): a randomised, placebo-controlled double-blind trial. Lancet 2011;377:741-50.
6. Anderson CS, Heeley E, Huang Y, et al. Rapid blood-pressure lowering in patients with acute intracerebral hemorrhage. N Engl J Med 2013;368:2355-65.
7. Willmot MR, Bath PMW. The potential of nitric oxide therapeutics in stroke. Expert Opinion Investigational Drugs 2003;12:455-70.
8. Willmot M, Murphy S, Leonardi-Bee J, Bath P. Systematic review of nitric oxide donors and L-arginine in experimental stroke: effects on infarct size and cerebral blood flow. CerebrovascDis 2003;16:S3 66 (abstract).
9. Butterworth RJ, Cluckie A, Jackson SHD, Buxton-Thomas M, Bath PMW. Pathophysiological assessment of nitric oxide (given as sodium nitroprusside) in acute ischaemic stroke. Cerebrovasc Dis 1998;8:158-65.
10. Bath PMW, Pathansali R, Iddenden R, Bath FJ. The effect of transdermal glyceryl trinitrate, a nitric oxide donor, on blood pressure and platelet function in acute stroke. Cerebrovasc Dis 2001;11:265-72.
11. Rashid P, Weaver C, Leonardi-Bee JA, Fletcher S, Bath FJ, Bath PMW. The effects of transdermal glyceryl trinitrate, a nitric oxide donor on blood pressure, cerebral and cardiac haemodynamics and plasma nitric oxide levels in acute stroke. J Stroke Cerebrovasc Dis 2003;13:143-51.
12. Willmot M, Ghadami A, Whysall B, Clarke W, Wardlaw J, Bath PM. Transdermal glyceryl trinitrate lowers blood pressure and maintains cerebral blood flow in recent stroke. Hypertension 2006;47:1209-15.
13. Ankolekar S FM, Renton C, Cox P, Sprigg N, Siriwardena AN, Bath PM. Feasibility of an ambulance-based stroke trial and safety of glyceryl trinitrate in ultra acute stroke: the 'Rapid Intervention with Glyceryl Trinitrate in Hypertensive Stroke Trial' (RIGHT, ISRCTN66434824). Stroke 2013.
14. Ankolekar S, Sare G, Geeganage C, et al. Determining the Feasibility of Ambulance-Based Randomised Controlled Trials in Patients with Ultra-Acute Stroke: Study Protocol for the "Rapid Intervention with GTN in Hypertensive Stroke Trial" (RIGHT, ISRCTN66434824). Stroke Res Treat 2012;2012:385753.
15. The ENOS Trial Investigators. Glyceryl trinitrate vs. control, and continuing vs. stopping temporarily prior antihypertensive therapy, in acute stroke: rationale and design of the Efficacy of Nitric Oxide in Stroke (ENOS) trial (ISRCTN99414122). International Journal of Stroke 2006;1:245-9.
16. Kobayashi A, Grabska K, Czlonkowska A, Gray LJ, Bath PMW. Efficacy of Nitric Oxide in Stroke - ENOS - a randomized trial - Characteristics of patients recruited in Poland. Polish Journal of Neurology and Neursurgery 2008;42:99-104.
17. Sare GM, Gray LJ, Wardlaw J, Chen C, Bath PMW, Investigators FtET. Is lowering blood pressure hazardous in patients with significant ipsilateral carotid stenosis and acute

ischaemic stroke? Interim assessment in the 'Efficacy of Nitric Oxide in Stroke' Trial. Blood Pressure Monitoring 2009;14:20-5.
18. Ankolekar S, Renton C, Bereczki D, et al. Effect of the neutral CLOTS 1 trial on the use of graduated compression stockings in the Efficacy of Nitric Oxide Stroke (ENOS) trial. J Neurol Neurosurg Psychiatry 2012.
19. Whynes DK, Sprigg N, Selby J, Berge E, Bath PM. Testing for differential item functioning within the EQ-5D. Med Decis Making 2013;33:252-60.
20. Ildiko S SS, Jurcau A, Simion A, Sisak E, Renton C, Ellender S, Bath P. The efficacy of nitric oxide in stroke (ENOS) Trial - where do we stand in Romania? Romanian Journal of Neurology 2012;XI:68.
21. Weir CJ, Lees KR. Comparison of stratification and adaptive methods for treatment allocation in an acute stroke clinical trial. StatMed 2003;22:705-26.
22. Bath P, Lindenstrom E, Boysen G, et al. Tinzaparin in acute ischaemic stroke (TAIST): a randomised aspirin-controlled trial. Lancet 2001;358:702-10.
23. Bamford J, Sandercock P, Dennis M, Burn J, Warlow C. Classification and natural history of clinically identifiable subtypes of cerebral infarction. Lancet 1991;337:1521-6.
24. The Optimising Analysis of Stroke Trials (OAST) Collaboration. Can we improve the statistical analysis of stroke trials? Statistical re-analysis of functional outcomes in stroke trials. Stroke 2007;38:1911-5.
25. Bath PM, Lees KR, Schellinger PD, et al. Statistical analysis of the primary outcome in acute stroke trials Stroke 2012;43:1171-8.
26. The Optimising Analysis of Stroke Trials (OAST) Collaboration. Should stroke trials adjust functional outcome for baseline prognostic factors? Stroke 2009;40:888-94.
27. Saver JL. Novel-end point analytic techniques and interpreting shifts across the entire range of outcome scales in acute stroke trials. Stroke 2007.
28. Savitz SI, Lew R, Bluhmki E, Hacke W, Fisher M. Shift analysis versus dichotomization of the Modified Rankin scale outcome scores in the NINDS and ECASS-II trials. Stroke 2007;38:3205-12.
29. Lees KR, Zivin JA, Ashwood T, et al. NXY-059 for acute ischemic stroke. New England Journal of Medicine 2006;354:588-99.
30. Shuaib A, Lees KR, Lyden P, et al. NXY-059 for the treatment of acute ischemic stroke. New England Journal of Medicine 2007;357:562-71.
31. Hacke W, Kaste M, Bluhmki E, et al. Thrombolysis with alteplase 3 to 4.5 hours after acute ischemic stroke. New England Journal of Medicine 2008;359:1317-29.
32. Rankin J. Cerebral vascular accidents in patients over the age of 60. 2. Prognosis. Scottish Medical Journal 1957;2:200-15.
33. Tilley BC, Marler J, Geller NL, et al. Use of a global test for multiple outcomes in stroke trials with application to the National Institute of Neurological Disorders and Stroke t-PA stroke trial. Stroke 1996;27:2136-42.
34. Intravenous Magnesium Efficacy in Stroke (IMAGES) Study Investigators. Magnesium for acute stroke (Intravenous Magnesium Efficacy in Stroke trial): randomised controlled trial. Lancet 2004;363:439.
35. Gray LJ, Ali M, Lyden PD, Bath PM. Interconversion of the National Institutes of Health Stroke Scale and Scandinavian Stroke Scale in acute stroke. J Stroke Cerebrovasc Dis 2009;18:466-8.
36. Graeb DA, Robertson WD, Lapointe JS, Nugent RA, Harrison PB. COMPUTED TOMOGRAPHIC DIAGNOSIS OF INTRAVENTRICULAR HEMORRHAGE - ETIOLOGY AND PROGNOSIS. Radiology 1982;143.
37. Morgan TC DJ, Spengler D, Lees KR, Aldrich C, Misha NK, Lane K, Quinn TJ, Deiner-Wet M, Weir CJ, Higgins P, Refferty M, Kinsley K, Ziai W, Awad I, Walters MR, Hanley D. The Modified Graeb Score: An enhanced Tool for Intraventricular Hemorrhage Measurement and Prediction of Functional Outcome. Stroke 2013;44:635-41.
38. Cheung K, Oemar M, Oppe M, Rabin R. EQ-5D User Guide: Basic information on how to use EQ-5D In. 2 ed. [http://www.euroqol.org:](http://www.euroqol.org) EuroQol Group; 2009.

39. Sandercock P, Wardlaw JM, Lindley RI, et al. The benefits and harms of intravenous thrombolysis with recombinant tissue plasminogen activator within 6 h of acute ischaemic stroke (the third international stroke trial [IST-3]): a randomised controlled trial. Lancet 2012;379:2352-63.
40. Gray LJ, Ali M, Lyden PD, Bath PMW, Collaboration ftVISTA. Interconversion of the National Institutes of Health Stroke Scale and Scandinavian Stroke Scale in acute stroke. Journal of Stroke and Cerebrovascular Diseases 2009;18:466-8.
41. Wardlaw J, Bath P, Sandercock P, et al. The NeuroGrid stroke examplar clinical trial protocol. International Journal of Stroke 2007;2:63-9.
42. Sare GM, Gray LJ, Wardlaw J, Chen C, Bath PMW, ENOS Trial Investigators. Is lowering blood pressure hazardous in patients with significant ipsilateral carotid stenosis and acute ischaemic stroke? Interim assessment in the 'Efficacy of Nitric Oxide in Stroke' Trial. Blood Pressure Monitoring 2009;14:20-5.
43. De Silva DA, Omar E, Manzano JJ, et al. Comparison of small volume infarcts of lacunar and non-lacunar etiologies. Int J Stroke 2013;8:E24-5.
44. Sandset EC MG, Bath PMW, Kjeldsen SE, Berge E: Scandinavian Candesartan Acute Stroke Trial (SCAST) Study Group. Relationship between change in blood pressure in acute stroke and risk of early adverse events and poor outcome. Stroke 2012;43:2108-14.
45. Gray LJ, Sprigg N, Rashid PA, Willmot M, Bath PMW. Effect of nitric oxide donors on blood pressure and pulse pressure in acute and sub-actue stroke. Journal of Stroke and Cerebrovascular Diseases 2006;15:245-9.
46. Weir NU, Sandercock PAG, Lewis SC, Signorini DF, Warlow CP, Group obotIC. Variations between countries in outcome after stroke in the International Stroke trial (IST). Stroke 2001;32:1370-7.
47. Bath P, Soerensen P, Lindenstrom E. Outcome after stroke varies between countries: data from the 'Tinzaparin in Acute Ischaemic Stroke Trial' (TAIST). Cerebrovasc Dis 2001;11 (suppl 4):7 (abstract).
48. Ali M, Atula S, Bath PMW, et al. Stroke outcome in clinical trial patients deriving from different countries. Stroke 2008.
49. Robinson TG, Potter JF, Ford GA, et al. Effects of antihypertensive treatment after acute stroke in the Continue or Stop Post-Stroke Antihypertensives Collaborative Study (COSSACS): a prospective, randomised, open, blinded-endpoint trial. Lancet Neurol 2010;9:767-75.
50. Bath PMW, Willmot M, Bath-Hextall FJ, Leonardie-Bee J. Nitric oxide donors (nitrates), L-arginine, or nitric oxide synthase inhibitors in acute ischaemic stroke Cochrane Database of Systematic Reviews 2002.
51. Geeganage C, Bath PM. Vasoactive drugs for acute stroke. Cochrane Database Syst Rev 2010:CD002839.
52. Geeganage C, Bath PMW. Interventions for deliberately altering blood pressure in acute stroke (Review). The Cochrane Library 2008.
53. Sheth KN, Cushing TA, Wendell L, et al. Comparison of hematoma shape and volume estimates in warfarin versus non-warfarin-related intracerebral hemorrhage. Neurocrit Care 2010;12:30-4.
54. Sandset EC, Murray GD, Bath PM, Kjeldsen SE, Berge E. Relation between change in blood pressure in acute stroke and risk of early adverse events and poor outcome. Stroke 2012;43:2108-14.
55. Perez I, Smithard DG, Davies H, Kalra L. Pharmacological treatment of dysphagia in stroke. Dysphagia 1998;13:12-6.
56. Gray LJ, Sprigg N, Bath PM, et al. Significant variation in mortality and functional outcome after acute ischaemic stroke between western countries: data from the tinzaparin in acute ischaemic stroke trial (TAIST). J Neurol Neurosurg Psychiartry 2006;77:327-33.
57. Lees KR, Bluhmki E, von Kummer R, et al. Time to treatment with intravenous alteplase and outcome in stroke: an updated pooled analysis of ECASS, ATLANTIS, NINDS, and EPITHET trials. Lancet 2010;375:1695-703.

58. Details of a prospective protocol for a collaborative meta-analysis of individual participant data from all randomized trials of intravenous rt-PA vs. control: statistical analysis plan for the Stroke Thrombolysis Trialists' Collaborative meta-analysis. Int J Stroke 2013;8:278-83.
59. Optimising the Analysis of Stroke Trials (OAST) Collaboration. Calculation of Numbers-Needed-to-Treat (NNT) in parallel group trials assessing ordinal outcomes: Case examples from acute stroke and stroke prevention International Journal of Stroke 2011;6:472-9.
